# Retinoschisin and novel Na/K-ATPase interaction partners Kv2.1 and Kv8.2 define a growing protein complex at the inner segments of mammalian photoreceptors

**DOI:** 10.1007/s00018-022-04409-9

**Published:** 2022-07-25

**Authors:** Verena Schmid, Alexander Wurzel, Christian H. Wetzel, Karolina Plössl, Astrid Bruckmann, Patricia Luckner, Bernhard H. F. Weber, Ulrike Friedrich

**Affiliations:** 1 Institute of Human Genetics, University of Regensburg, Franz-Josef-Strauss-Allee 11, 93053 Regensburg, Germany; 2 Department of Psychiatry and Psychotherapy, University of Regensburg, Franz-Josef-Strauss-Allee 11, 93053 Regensburg, Germany; 3 Institute of Biochemistry, Genetics and Microbiology, Protein Mass Spectrometry Group, University of Regensburg, Universitätsstraße 31, 93053 Regensburg, Germany; 4 Institute of Clinical Human Genetics, University Hospital Regensburg, Franz-Josef-Strauss-Allee 11, 93053 Regensburg, Germany

**Keywords:** X-linked juvenile retinoschisis, Retinoschisin, RS1, Voltage-gated potassium channel, Kv2.1, Kv8.2, Retinal Na/K-ATPase

## Abstract

The *RS1* gene on Xp 22.13 encodes retinoschisin which is known to directly interact with the retinal Na/K-ATPase at the photoreceptor inner segments. Pathologic mutations in *RS1* cause X-linked juvenile retinoschisis (XLRS), a hereditary retinal dystrophy in young males. To further delineate the retinoschisin-Na/K-ATPase complex, co-immunoprecipitation was performed with porcine and murine retinal lysates targeting the ATP1A3 subunit. This identified the voltage-gated potassium (Kv) channel subunits Kv2.1 and Kv8.2 as direct interaction partners of the retinal Na/K-ATPase. Colocalization of the individual components of the complex was demonstrated at the membrane of photoreceptor inner segments. We further show that retinoschisin-deficiency, a frequent consequence of molecular pathology in XLRS, causes mislocalization of the macromolecular complex during postnatal retinal development with a simultaneous reduction of Kv2.1 and Kv8.2 protein expression, while the level of retinal Na/K-ATPase expression remains unaffected. Patch-clamp analysis revealed no effect of retinoschisin-deficiency on Kv channel mediated potassium ion currents in vitro. Together, our data suggest that Kv2.1 and Kv8.2 together with retinoschisin and the retinal Na/K-ATPase are integral parts of a macromolecular complex at the photoreceptor inner segments. Defective compartmentalization of this complex due to retinoschisin-deficiency may be a crucial step in initial XLRS pathogenesis.

**Supplementary Information:**

The online version contains supplementary material available at 10.1007/s00018-022-04409-9.

## Introduction

X-linked juvenile retinoschisis (XLRS, OMIM #312700) is a hereditary retinal dystrophy affecting males with an estimated prevalence between 1:5.000 and 1:20.000. Characteristic features of XLRS are splitting of the inner retinal layers, and defective signal transmission from photoreceptors to bipolar cells [1, 2]. Pathologic mutations in the *RS1* gene, which is specifically expressed in photoreceptors and bipolar cells of the retina, as well as pinealocytes of the pineal gland [3, 4] were shown to be causative of XLRS [5].

The *RS1* gene encodes a secreted protein termed retinoschisin which is anchored to the retinal plasma membrane upon binding to the retinal Na/K-ATPase [6, 7]. Na/K-ATPases are plasma membrane-spanning ion pumps composed of two subunits [8]: a catalytic alpha-subunit with four known isoforms ATP1A1 to ATP1A4 and a regulatory beta-subunit with three known isoforms ATP1B1 to ATP1B3 [9, 10]. The alpha and beta isoforms are tissue-specific and depending on the respective combination of the isoforms reveal distinctive features in catalytic activity, ligand binding, and regulatory function [9]. In the retina, ATP1A3 and ATP1B2 are the predominant isoforms [11], and thus the enzyme complex composed of ATP1A3 and ATP1B2 is referred to as the “retinal Na/K-ATPase”. While little is known about the retinal Na/K-ATPase, other Na/K-ATPases, e.g. those that are formed by ATP1A1 and any other beta-subunit, are mainly expressed in heart or liver and are well-studied [12]. They are reported to function not only as an ion pump, essential for maintaining cellular ion homeostasis, but also as a protein docking station for an increasing number of proteins, thus enabling the formation of specific plasma membrane microdomains [10, 13]. Specifically, Na/K-ATPases were reported to assemble with intracellular signal transducers such as Src (Src protooncogene, nonreceptor tyrosine kinase), phospholipase C, or the Inositol trisphosphate (IP3)-receptor, leading to the formation of so-called “signalosome complexes” [10, 13] Isoforms ATP1A1 and ATP1A2 further form macromolecular complexes with other ion channels or transporters, such as aquaporin or the Na/Ca exchanger (NCX) [10]. Scaffolding proteins like caveolin or ankyrin B (AnkB) were reported to play essential roles in the formation of these membrane microdomains [10, 14].

To further elucidate the molecular processes underlying XLRS pathogenesis, we aimed to identify additional components of the retinoschisin-Na/K-ATPase complex and explored the consequences of retinoschisin-deficiency on the integrity of such a complex. We performed co-immunoprecipitation analyses targeting the ATP1A3 isoform of the retinal Na/K-ATPase in porcine and murine retinal lysates followed by an in depth-characterization of newly identified interaction partners, namely the voltage-gated potassium (Kv) channel subunits Kv2.1 and Kv8.2.

## Materials and methods

### Animal models

Rs1h^−/y^ (Rs1^tm1Web^) mice were kept on a C57BL/6 J background for more than 40 generations. Mice were housed under specific pathogen-free barrier conditions at the Central Animal Facility of the University of Regensburg, in strict compliance with NIH guidelines. Mice were sacrificed by cervical dislocation at different postnatal days (P), specifically at P4, P7, P10, P14, P18, P21, or P30. Porcine eyes were obtained fresh from a local slaughterhouse.

### Cell culture

Y-79 (RRID:CVCL_1893**,** ATCC; LGC Standards GmbH, Wesel, Germany) and Hek293 cells (“Hek293 EBNA cells” RRID:CVCL_0045, Invitrogen, Carlsbad, CA, USA) were cultivated as described by Biasella and colleagues [15]. For the cultivation of Hek293 cells stably expressing recombinant retinoschisin, hygromycin (150 μg/ml) was added to the standard Hek293 cultivation medium.

### Expression constructs

The coding sequences of *KCNB1* (NM_004975.4) and *KCNV2* (NM_133497) were amplified from cDNA of Y-79 (oligonucleotide primer sequences, see Supplementary Table S3) and cloned into the KpnI/NotI site of the pcDNA3.1 vector (Invitrogen, Thermo Fisher Scientific, Waltham, MA, USA). Generation of expression constructs for untagged retinoschisin (NM_000330.4) is described by Friedrich and colleagues [6], for the expression of Myc-tagged retinoschisin by Plössl and colleagues [16], and for the bicistronic expression of ATP1A3 (NM_152296.4) and ATP1B2 (NM_001678.4) in [17].

### Transfection

For heterologous expression of Kv channels (Kv2.1/Kv8.2) or the retinal Na/K-ATPase (ATP1A3/ATP1B2), Hek293 cells were transfected with Mirus Bio *Trans*IT-LTI Transfection Reagent (Thermo Fisher Scientific). For heterologous expression of retinoschisin, Hek293 cells were transfected using the calcium-phosphate method [18].

### Isolation of cell-surface proteins

48 h after transfection, cell-surface expression of heterologously expressed proteins was analyzed using the Pierce™ Cell-Surface Protein Isolation Kit (Thermo Fisher Scientific). According to the manufacturer’s instructions, cell-surface proteins were biotinylated, purified and subsequently analyzed by western blot analysis.

### Binding of retinoschisin to transfected Hek293 cells

Retinoschisin binding to Hek293 cells heterologously expressing the retinal Na/K-ATPase, Kv2.1, Kv8.2, or Kv2.1 plus Kv8.2 was performed as described [6, 16, 17, 19], but with a prolonged incubation time of 1 h. The supernatant of Hek293 cells stably secreting recombinant retinoschisin was used as input.

### SDS-PAGE and western blot analysis

SDS-PAGE and western blot analysis were performed as previously described in [6, 20]. Detailed information of primary antibodies is given in Supplementary Table S4. Prior to the use in the experiments, antibodies were tested on retinal lysates and Hek293 cells for antibody specificity (Supplementary Fig. S10a). To allow simultaneous staining of several proteins after SDS-PAGE and western blotting, specifically of samples with limited availability, e.g. after co-immunoprecipitation, PVDF membranes were sectioned after western blotting followed by immunostaining with an array of individual antibodies. While figures presented in the results section show cropped blots, full-sized original stainings are given in Supplementary Fig. S10b–e). Densitometric analysis was performed with the Image Studio software (LI-COR Biosciences, Lincoln, NE, USA).

### Co-Immunoprecipitation analysis

Protein A Sepharose beads (Sigma-Aldrich, St. Louis, MO, USA) coupled to antibodies, were prepared as follows: 50 µl of the beads were washed three times with 1 ml of phosphate-buffered saline (PBS) and spun down for 2 min at 2000 rpm at 4 °C. After discarding the supernatant, beads were conjugated with the respective antibodies, beads and antibody solution (2 µg in 100 µl PBS) were mixed and rotated at 4 °C for 1 h followed by centrifugation for 2 min at 2000 rpm at 4 °C. Subsequently, the supernatant was discarded, and antibody-coupled beads were mixed with the input.

Retinal lysates were prepared as follows: for each co-immunoprecipitation experiment, 10 mg of porcine retinae was dissolved in 3 ml of 10 mM Cholamidopropyl-dimethylammonio-1-propansulfonat (CHAPS) in Tris-buffered saline (TBS) followed by sonification time at 40% for of 45 s. Alternatively, four retinae of wildtype mice (P18) were homogenized in 1 ml of 10 mM CHAPS in TBS and sonicated for 10 s at 40%. The suspensions were rotated at 4 °C for 1 h and cell debris was removed by centrifugation for 20 min at 13,000 rpm and 4 °C. The supernatant containing the retinal lysate was collected and used as “input”.

Input (3 ml of porcine lysate or 1 ml of murine lysate) was added to the antibody-coupled beads. After overnight rotation at 4 °C, the beads were spun down for 2 min at 2000 rpm and 4 °C. 50 µl of the supernatant was collected as “flow through” fraction and the rest was discarded. To remove unbound protein, beads were washed five times with 1 ml 10 mM CHAPS in TBS (centrifugation as described at each step) and 100 µl of the last wash fraction (“wash”) was collected. Precipitated proteins were eluted with 1 × Laemmli-buffer (Laemmli 1970), and input, flow through, and wash fractions were mixed with 5 × Laemmli-buffer. Samples were denatured at 93 °C for 10 min and subjected to Coomassie Blue staining (porcine retinal lysates) or western blot analysis (murine lysates). In co-immunoprecipitation experiments the flow through fraction was frequently similar in intensity to the input. This suggests an excess of protein exceeding the binding capacity of the beads and leading to a minor decrease of the total protein amount after co-immunoprecipitation (and thus similar flow through intensities). Moreover, immunolabelling signals in the precipitate fractions were frequently weak, especially for ATP1A3, Kv2.1, and Kv8.2. Due to inherent properties of membrane proteins [21], co-immunoprecipitations of membrane complexes are challenging. Also, sensitivities of antibodies vary widely. As a consequence, quantitation based on immuno-stainings is rather speculative and was avoided in the present study.

### Mass spectrometry

Proteins were separated on a precast 4–12% NUPAGE Bis–Tris gel (Invitrogen) and stained with Coomassie Blue. Gel lanes were cut into stripes, washed with 50 mM NH_4_HCO_3_ and lyophilized. After a reduction/alkylation treatment and additional washing steps, proteins were *in gel* digested with trypsin (Trypsin Gold, mass spectrometry grade, Promega) overnight at 37 °C. The resulting peptides were sequentially extracted with 50 mM NH_4_HCO_3_ and 50 mM NH_4_HCO_3_ in 50% acetonitrile. After lyophilization, peptides were reconstituted in 20 µl 1% TFA and separated by reversed-phase chromatography. An UltiMate 3000 RSLCnano System (Thermo Fisher Scientific, Dreieich) equipped with a C18 Acclaim Pepmap100 preconcentration column (100 µm i.d. × 20mm, Thermo Fisher Scientific) and an Acclaim Pepmap100 C18 nano column (75 µm i.d. × 250 mm, Thermo Fisher Scientific) was operated at flow rate of 300 nl/min and a 60 min linear gradient of 4% to 40% acetonitrile in 0.1% formic acid. The LC was online-coupled to a maXis plus UHR-QTOF System (Bruker Daltonics) via a CaptiveSpray nanoflow electrospray source. Acquisition of MS/MS spectra after collision-induced dissociation (CID) fragmentation was performed in data-dependent mode at a resolution of 60,000. The precursor scan rate was 2 Hz processing a mass range between m/z 175 and m/z 2000. A dynamic method with a fixed cycle time of 3 s was applied via the Compass 1.7 acquisition and processing software (Bruker Daltonics).

MaxQuant Version 2.0.3.0 [22] was used for searches against the Uni-Prot *Sus scrofa* database with the following parameters: enzyme specificity trypsin with two missed cleavages and cleavage before proline allowed, precursor tolerance 0.02 Da and MS/MS tolerance 0.04 Da. Deamidation of asparagine and glutamine, oxidation of methionine, Nt-Acetylation, carbamidomethylation or propionamide modification of cysteine were set as variable modifications. Calculation of iBAQ values was activated and protein and peptide identifications were filtered at a false discovery rate (FDR) < 1%. For complex stoichiometry estimation iBAQ value ratios were calculated using MS Excel (Version 2018).

### RNA expression analysis

RNA was isolated from murine retinae of wildtype and retinoschisin-deficient mice using the PureLink™ RNA Micro Kit (Invitrogen), according to the manufacturers’ protocols. One microgram of total RNA was transcribed into cDNA using Random Hexamer Primers (Thermo Fisher Scientific) and the RevertAid M‐MuLV Reverse Transcriptase (Fermentas, St Leon‐Rot, Germany) according to the manufacturer's instructions. Quantitative real‐time PCR was performed in technical triplicates using KiCqStart® probe assays from Sigma Aldrich (Supplementary Table S5) and the QuantStudio5 System (Thermo Fisher Scientific). Results were analyzed by applying the ΔΔCt method for relative quantification [23].

### Immunocytochemical analyses

Glass cover slips (12 mm) were coated with Poly-L-Lysin for 1 h at 37 °C. Hek293 cells were seeded on coated cover slips, transfected with the different expression constructs, and cultured for 24 h. Immunocytochemical analysis was performed as described by Brandl and colleagues [24] with the following modifications: Primary antibody stainings (Supplementary Table S4) were shortened to 4 h and secondary antibody stainings together with 4′,6-diamidino-2-phenylindol (DAPI, 1:1000, Molecular Probes, Leiden, The Netherlands) to 30 min. Confocal microscopic images were taken with an Olympus Fv3000 confocal laser scanning microscope (Olympus Europa SE & Co. KG, Hamburg, Germany) at 60 × magnification.

### Immunohistochemical analyses

Murine eyes (wildtype, retinoschisin-deficient or Atp1b2-deficient) were enucleated, fixed, embedded, cryosectioned, and immunolabeled as described by Friedrich and colleagues [6]. Antibodies used for immunolabeling are given in Supplementary Table S4. Sections were counterstained with DAPI. Images were taken with an Olympus Fv3000 confocal laser scanning microscope (Olympus Europa SE & Co. KG, Hamburg, Germany) at 20 × or 60 × magnification. For Co-/Localization analysis at 60x, confocal image stacks (3 slides, distance 0.26 µm) were processed via the Wiener 3D-Deconvolution tool provided by the Olympus CellSens image processing software (CellSens Dimension).

### Protein expression analysis

For each replicate, the two retinae of a single mouse (wildtype or retinoschisin-deficient, postnatal stages depicted in Fig. 5) were sonicated in 100 µl of PBS followed by the addition of 25 µl of 5 × Laemmli-buffer. Samples were heated at 93 °C for 5 min and subjected to western blot analysis.

### Purification of recombinant retinoschisin

Purification of recombinant retinoschisin from supernatant of Hek293 cells heterologously expressing Myc-tagged retinoschisin was performed as described before [17, 19, 20]. Supernatant of Hek293 cells transfected with the empty pCDNA3.1™ expression vector (Thermo Fisher Scientific) was subjected to the identical purification procedure and served as control.

### Electrophysiology

Glass cover slips (18 mm) were coated with concanavalin-A (0.5% in 1 M NaCl) for 2 h at 37 °C and then rinsed with distilled water. Y-79 cells were seeded on the coated cover slips and cultured for 24 h in medium containing purified retinoschisin (1 µg/ml) or the same volume of control eluate. Electrophysiological measurements of potassium channel currents were performed as described by Gomez M del P and colleagues [25], with slight modifications: Patch pipettes had a tip resistance of 3–4 MΩ and were filled with intracellular solution containing 130 mM KCl, 4 mM Na_2_ATP, 4 mM MgCl_2_, 10 mM HEPES, and 1 mM EGTA. Cells were bathed in extracellular solution containing 130 mM NaCl, 5 mM KCl, 10 mM CaCl_2_, 2 mM MgCl_2_, 10 mM HEPES. Single cells (without contact to other cells) were voltage-clamped at a holding potential of − 60 mV with voltage steps between − 60 and 80 mV in 10 mV increments. The whole-cell voltage-clamp recordings were performed by using an EPC-10 USB amplifier on an Olympus IX73 microscope (Olympus Europa SE & Co. KG, Hamburg, Germany) and the Patchmaster Software (HEKA, Lambrecht, Germany). To test whether the measured outward current is generated by potassium channels, KCl in intracellular and extracellular solutions was replaced by CsCl or the specific Kv2 channel inhibitor citalopram (50 µM and 200 µM, respectively) was added to the extracellular solution.

### Statistical analyses

Normality of the data was assessed by the Shapiro–Wilk normality test. Data not following a Gaussian distribution were analyzed using Mann–Whitney-*U* test (2 experimental groups). Data following a Gaussian distribution were analyzed using Student’s *t*-test (2 experimental groups). Statistical analyses were performed using the “XLSTAT add-in” software.

## Results

### Kv2.1 and Kv8.2 are direct interaction partners of the retinal Na/K-ATPase

To identify new interaction partners of the retinal Na/K-ATPase, we initially performed co-immunoprecipitation experiments with porcine retinal lysates targeting ATP1A3, followed by mass spectrometric analysis of precipitated proteins. Co-immunoprecipitation experiments with anti-6*His-tag antibodies were included as a negative control. A comprehensive list of proteins detected by mass spectrometry in the precipitate fractions of both experimental approaches (three independent biological replicates for co-immunoprecipitation experiments targeting ATP1A3 or control) is given in Supplementary Table S1 and S2. ATP1A3 revealed the highest abundance reflected by an intensity-based absolute quantification (iBAQ) value. ATP1B2 and retinoschisin were also detected albeit at lower levels (Supplementary Table S1). This demonstrates the capacity of the co-immunoprecipitation approach to precipitate ATP1A3 interacting proteins, like ATP1B2, and proteins attached to ATP1A3 binding proteins, like the ATP1B2 binding partner retinoschisin.

A candidate prioritization was done to select suitable proteins for further analysis. From a list of proteins exclusively detected or at least fivefold enriched in the ATP1A3-co-immunoprecipitation compared to control for all three replicates (Supplementary Table S1), candidates of interest were selected. To further refine the potential list of candidates, an extensive literature search was done focusing on proteins of the retina localizing to the inner segment (IS) of the photoreceptors, close to the retinal Na/K-ATPase). This focused our interest on Kv channel subunits Kv2.1 and Kv8.2, as both proteins have been localized to the photoreceptor IS membrane [26–29] and are known to play a predominant role in regulating phototransduction, intracellular signaling and retinal integrity [28, 30–33], all processes associated with XLRS pathology.

Stoichiometry of the ATP1A3 complex protein components chosen for further analyses was determined by calculating the ratios of the intensity based absolute quantification (iBAQ) values of ATP1A3 and the respective candidate proteins. The data revealed sub-stoichiometric amounts of Kv2.1 and Kv8.2 compared to ATP1A3 in the precipitate fractions. Detailed information on the stoichiometry, iBAQ values and sequence coverage of the ATP1A3 interaction partners ATP1B2, retinoschisin, Kv2.1, and Kv8.2 is given in Supplementary Table S2.

**Fig. 1:**
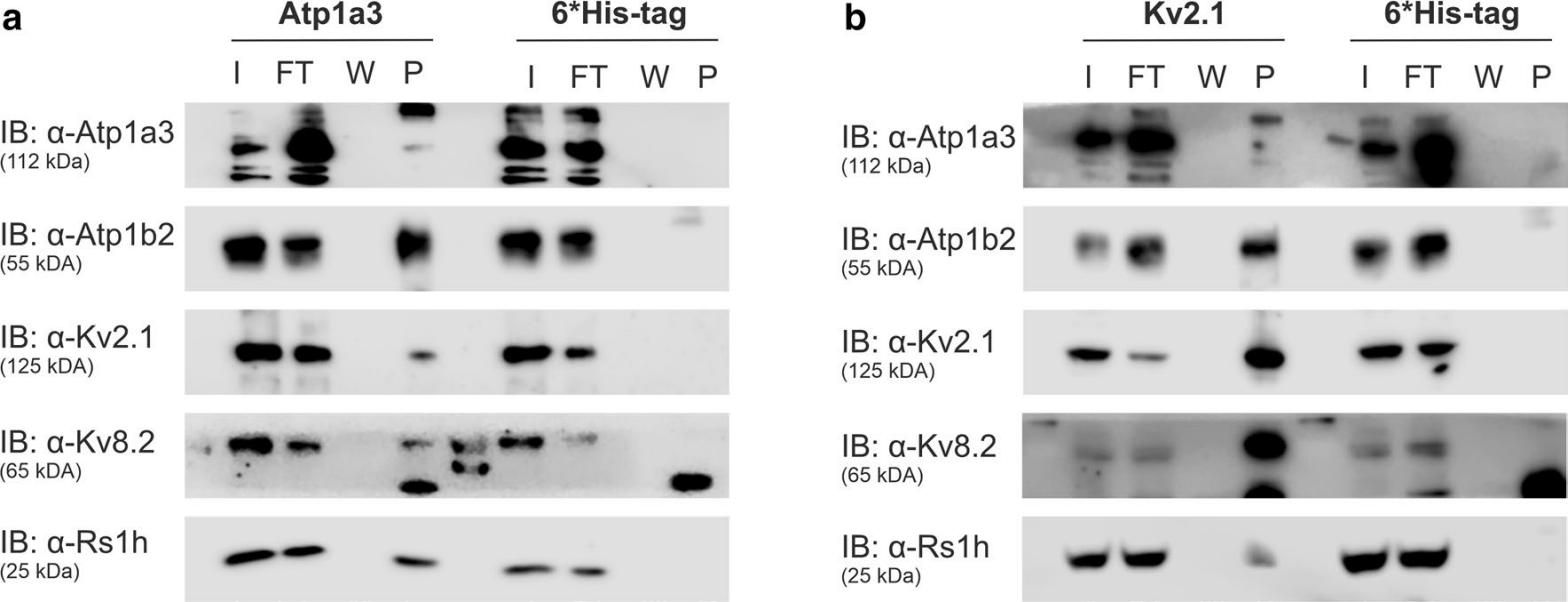
Kv channels Kv2.1 and Kv8.2 are binding partners of the retinoschisin-Na/K-ATPase complex in the murine retina. (**a** and **b**) Co-immunoprecipitation from murine retinal lysates with antibodies against Atp1a3 (**a**), Kv2.1 (**b**) and 6*His-tag as control (**a, b**). Samples of input (I), flow through (FT), the last washing fraction (W) and precipitate (P, contains co-immunoprecipitated proteins) were stained with antibodies against Atp1a3, Atp1b2, Kv2.1, Kv8.2, and retinoschisin

To confirm the interaction of ATP1A3 and the Kv channel subunits Kv2.1 and Kv8.2 in an independent approach, we performed co-immunoprecipitation with antibodies against Atp1a3 in murine retinal lysates followed by western blot analysis. Again, Kv2.1 and Kv8.2 were precipitated with Atp1a3, together with Atp1b2 and retinoschisin (Fig. 1a). As a control, experiments with anti-6*His-tag antibodies failed to detect any of these proteins in the precipitate (Fig. 1a). Reverse co-immunoprecipitation experiments were performed targeting Kv2.1 (Fig. 1b) or Kv8.2 (Supplementary Fig. S1a). Both approaches identified the two subunits of the retinal Na/K-ATPase (Atp1a3 and Atp1b2), and retinoschisin. Again, control experiments with anti-6*His-tag antibodies failed to identify these proteins in the precipitate (Fig. 1b, Supplementary Fig. S1a).

To demonstrate specificity of the approach chosen, we tested direct interaction of specific Na/K-ATPase isoforms to Src known to specifically interact with subunit Atp1a1 [34–36], but not with subunit Atp1a3 [37]. As expected, Src was solely found in co-immunoprecipitations with antibodies against Atp1a1, while retinoschisin was detected in co-immunoprecipitation fraction targeting Atp1a3 but not Atp1a1 (Supplementary Fig. S1b and c).

### Kv2.1, Kv8.2, and the retinoschisin-Na/K-ATPase complex are localized in the plasma membrane of photoreceptor inner segments

**Fig. 2:**
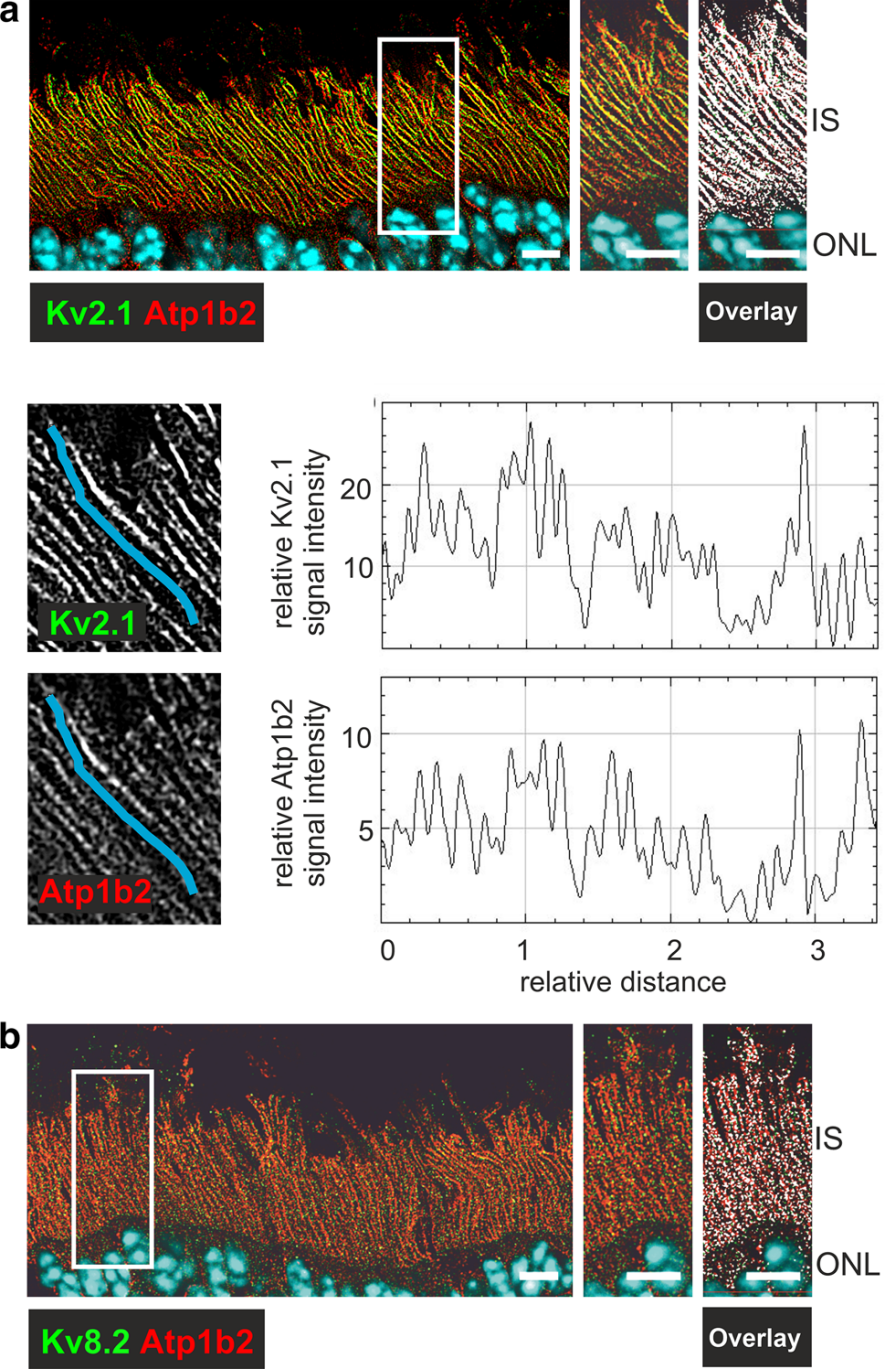
The retinal ATPase and the Kv channels show a similar distribution along the photoreceptor inner segment membranes of the murine retina. **a** and **b** Immunohistochemical stainings with antibodies against Kv2.1 (**a,** upper panel) or Kv8.2 (**b**) (green signals) and Atp1b2 (red signals, respectively). (**a**, middle and lower panel) Close-up images showing a Kv2.1 / Atp1b2 positive IS (marked blue) with overlapping signals. Representative line-scans along the inner segment membrane (for exact position see Supplementary Fig. S2c) revealing overlapping signal intensity profiles of anti-Kv2.1 and anti-Atp1b2 stainings. Scale bars: 5 µm; IS, inner segments; ONL, outer nuclear layer

Next, we investigated (co-)localization of the retinal Na/K-ATPase and Kv channel subunits Kv2.1 and Kv8.2 at the photoreceptor IS via immunohistochemistry of murine retinal cryosections. Consistent with the current literature [26–29, 38], the retinal Na/K-ATPase, retinoschisin and Kv2.1 were localized to the plasma membrane of the IS. Kv8.2 signals had a punctuate appearance, and, while labelling of the IS still is obvious, membrane localization could not be confirmed in this experiment. (Supplementary Fig. S2a, with close up shown in Supplementary Fig. S2b). To further determine co-localization of the retinal Na/K-ATPase and the Kv channel, simultaneous immunostaining of the Kv channel subunits with retinal ATPase subunit Atp1b2 was performed (Fig. 2). We observed an extensive overlap of Kv2.1 with Atp1b2 signals (Fig. 2a). Line-scan profiles of the co-stained Atp1b2 and Kv2.1 protein species along the IS membrane (Fig. 2a, Supplementary Fig. S2c and d reveal a largely overlapping intensity profile of Atp1b2 and Kv2.1 signals, suggesting a similar distribution of both proteins along the IS membrane. Kv8.2 was also established to overlap with Atp1b2 signals (Fig. 2b).

The localization of the complex partners was additionally analyzed in Atp1b2-deficient mice. These mice develop normally but display motor coordination deficits and retinal photoreceptor degeneration starting around postnatal day (P9) [39]. In Atp1b2-deficient mice from P10 to P18, no signals for both retinal Na/K-ATPase subunits were observed in the photoreceptor IS and only residual signals of Atp1a3 at the level of the outer plexiform layer (OPL) (Supplementary Fig. S3). In contrast, the immunohistochemical staining of wildtype murine retinae revealed strong Atp1a3 and Atp1b2 signals in photoreceptor IS and in the synaptic contacts of the OPL and inner plexiform layer (IPL) with increasing intensity during retinal maturation from P10 to P18 (Supplementary Fig. S3). Kv2.1 and Kv8.2 immunoreactivity was detected in photoreceptor IS of wildtype retinae with more prominent signals associated with advancing developmental age (Supplementary Fig. S4). Noteworthy, Atp1b2-deficiency causes a strong reduction in the immunolabeling of both Kv channels in Atp1b2-deficient mice in contrast to wildtype mice at all postnatal stages analyzed (Supplementary Fig. S4). Overall, Atp1b2-deficient retinae showed a more uniform staining pattern across the retinal layers and no defined labeling of the photoreceptor IS compared to wildtype retinae.

### Retinoschisin does not bind directly to Kv2.1 and Kv8.2

**Fig. 3:**
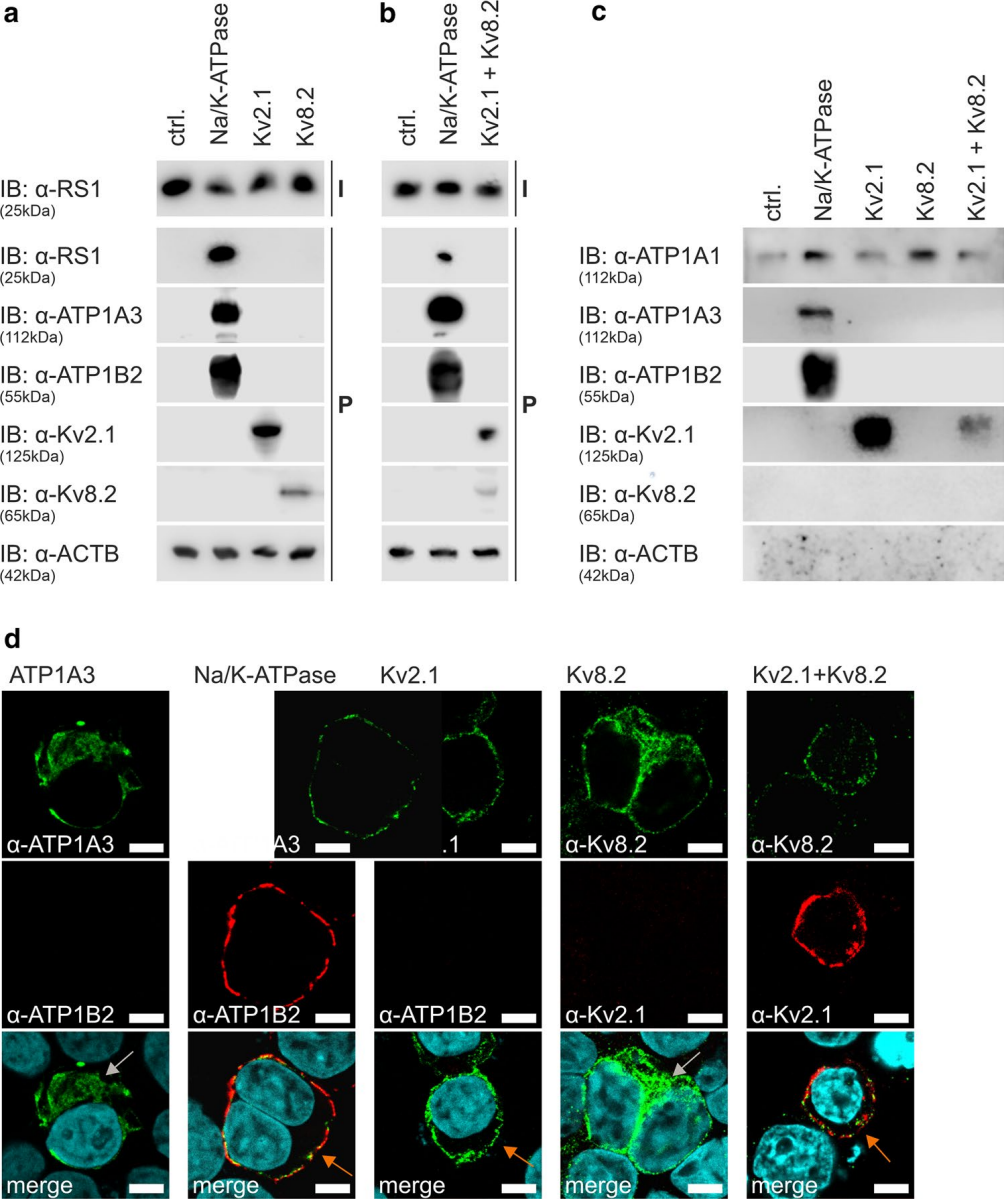
Kv2.1 and Kv8.2 do not directly bind to retinoschisin. **a** and **b** Retinoschisin binding to Hek293 cells heterologously expressing the retinal Na/K-ATPase (“Na/K-ATPase”), Kv2.1, Kv8.2 (**a**), or Kv2.1 plus Kv8.2 (**b**) was analyzed via western blot analysis (immunoblot, “IB” against retinoschisin, ATP1A3, ATP1B2, Kv2.1, Kv8.2 and ACTB). I = Retinoschisin-Input. P = Hek293 cells pellets after incubation with retinoschisin and intensive washing. **c** Cell-surface expression of ATP1A3, ATP1B2, Kv2.1, and Kv8.2 in Hek293 cells was analyzed by western blot analysis using antibodies against ATP1A3, ATP1B2, Kv2.1 and Kv8.2. The ATP1A1 and ACTB immunoblots were performed as controls for cell-surface or intracellular proteins, respectively. Hek293 cells transfected with empty expression vector (“ctrl.”) served as negative control. **d** Plasma membrane associated localization of ATP1A3, ATP1B2, Kv2.1, and Kv8.2 was analyzed after heterologous expression in Hek293 cells by immunocytochemical staining using antibodies against ATP1A3, ATP1B2, Kv2.1 and Kv8.2. Grey arrows indicate intracellular staining and orange arrows plasma membrane associated localization. Scale bars: 5 µm

To gain insight into the assembly of the Na/K-ATPase macromolecular complex, we examined whether retinoschisin, presenting multiple ligand binding sites in its hexadecameric conformation [40, 41], can directly bind Kv2.1 or Kv8.2 and might thus serve as a mediator between the retinal Na/K-ATPase and the Kv channels. Consequently, the binding capacity of externally added retinoschisin to the two endogenously expressed Kv subunits was tested in an established retinoschisin binding assay in Hek293 cells [6, 16, 20]. Binding of retinoschisin to the heterologously expressed retinal Na/K-ATPase served as a positive control [6, 16, 20]. While Kv.2.1 can form functional homotetramers, Kv8.2 only builds heterotetramers with other Kv channel subunits, such as Kv2.1 [42, 43]. Hence, overexpression of Kv2.1 and Kv8.2 alone, and in combination was done to allow homotetramer and heterotetramer formation, respectively. Retinoschisin bound to Hek293 cells heterologously expressing the retinal Na/K-ATPase, but not to cells transfected with an empty expression vector serving as negative control (Fig. 3a). Also, no binding was observed in Hek293 cells heterologously expressing Kv2.1, Kv8.2, or Kv2.1 plus Kv8.2 (Fig. 3a and b). To demonstrate cell-surface localization of the heterologously expressed proteins, we applied cell-surface protein biotinylation and immunocytochemical analysis of transfected Hek293 cells. While the retinal Na/K-ATPase subunits ATP1A3 and ATP1B2, as well as Kv2.1 were successfully detected in the cell-surface protein fraction (Fig. 3c), Kv8.2 was not, neither after single expression nor after co-expression with its essential partner protein Kv2.1. Immunocytochemical analysis of Hek293 cells expressing ATP1A3 alone revealed an intracellular localization of ATP1A3 (Fig. 3d, left column, grey arrow), contrary to Hek293 cells transfected with a bicistronic expression vector harboring ATP1A3 and ATP1B2, where the retinal Na/K-ATPase was localized at the plasma membrane (Fig. 3d, second column, orange arrow). A similar membrane localization was observed for Kv2.1 (Fig. 3d, third column, orange arrow) but not for Kv8.2, where an intracellular localization was detected (Fig. 3d, fourth column, grey arrow). Hek293 cells heterologously expressing Kv2.1 and Kv8.2 revealed membrane localization and colocalization of Kv channel subunits Kv2.1 and Kv8.2 (Fig. 3d, right column, orange arrow).

### Retinoschisin-deficiency leads to defective distribution of the retinal Na/K-ATPase and its interaction partners Kv2.1 and Kv8.2 in the developing retina of retinoschisin-deficient mice

Earlier analyses revealed that retinoschisin-deficiency affects the correct localization of the retinal Na/K-ATPase during murine retinal development starting at around P14 [6]. Here, we investigated the effect of retinoschisin-deficiency on the localization of the newly identified complex partners of the retinal Na/K-ATPase, Kv2.1 and Kv8.2, during early murine development (P4–P30). To quantify the localization of the complex partners, we measured signal intensity in murine retinal cryosections from wildtype and retinoschisin-deficient mice in IS and outer nuclear layers (ONL) and calculated the r(IS/ONL) ratio.

**Fig. 4:**
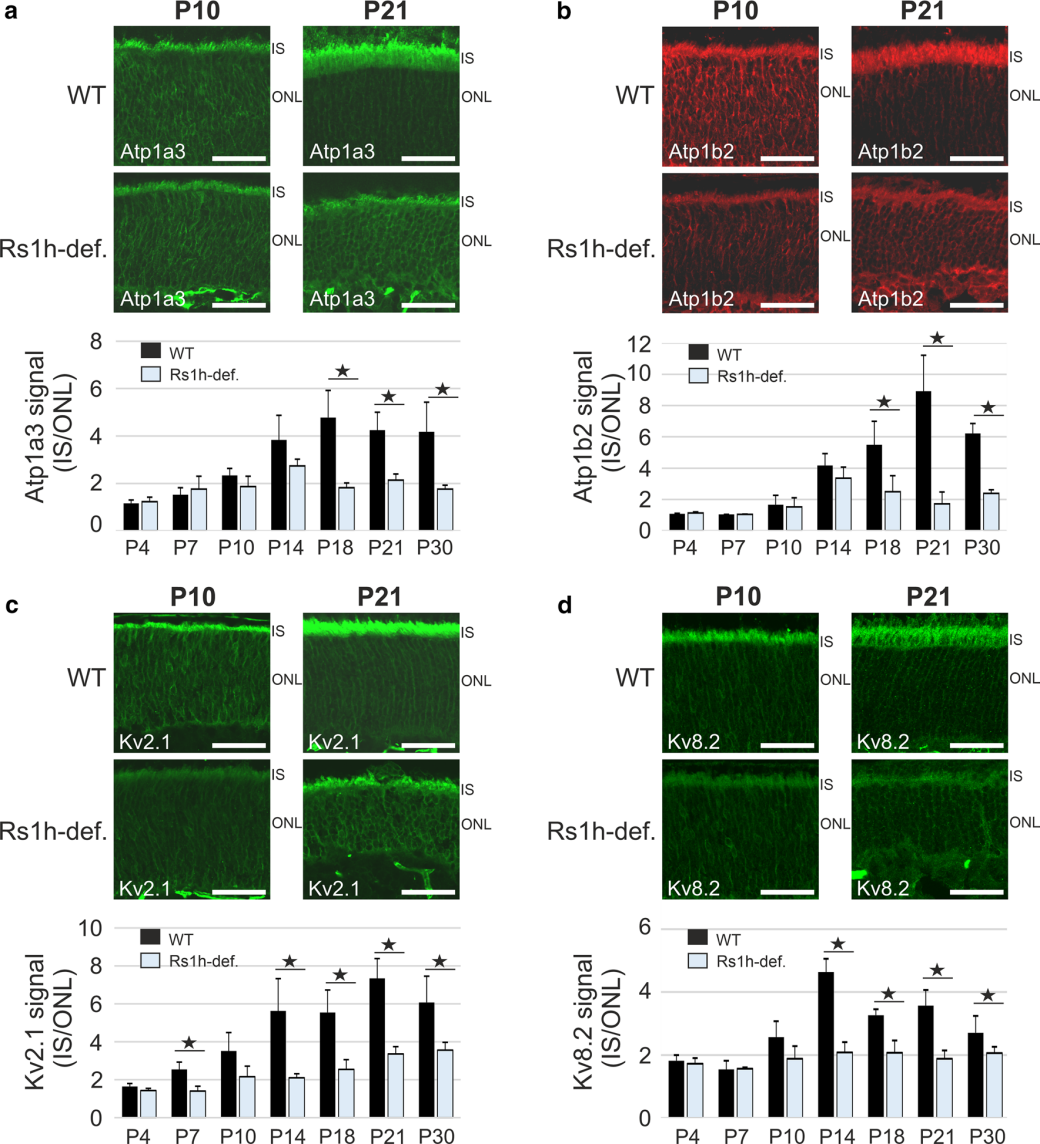
Retinoschisin is required for the localization of the retinal Na/K-ATPase as well as of Kv2.1 and Kv8.2 in the retina. (**a**–**d,** upper panel) Eye cup cryosections from wildtype (WT) and retinoschisin-deficient (Rs1h-def.) mice at P10 (left) and P21 (right) were subjected to confocal microscopy (20 × magnification) after immunohistochemistry with antibodies against Atp1a3 (**a**), Atp1b2 (**b**), Kv2.1 (**c**) and Kv8.2 (**d**). Scale bars: 40 µm; IS, inner segments; ONL, outer nuclear layer (**a – d,** lower panel) Atp1a3 (**a**), Atp1b2 (**b**), Kv2.1 (**c**) and Kv8.2 (**d**) signals in the inner segments and outer nuclear layer were quantified using ImageJ. Data show the ratio of signal intensity from inner to outer segments r(IS/ONL), given as mean ± SD of five biological replicates. *P < 0.05, two-tailed Student’s t-test

The distribution of the retinal Na/K-ATPase subunits Atp1a3 and Atp1b2 in the IS and the ONL was similar in wildtype and retinoschisin-deficient murine retinae from P4 to P10 (Fig. 4a and b, upper panels; Supplementary Figs. S5 and S6). At these stages the r(IS/ONL) ratio of Atp1a3 and Atp1b2 was around 1 at P4 and around 2 at P10 in wildtype and retinoschisin-deficient retinae (Fig. 4a and b, lower panels). From P14 onward, there was a strong accumulation of Atp1a3 and Atp1b2 in the IS from wildtype mice, but not from retinoschisin-deficient mice. The r(IS/ONL) values were statistically significant from P18 onward for Atp1a3 (r(IS/ONL) = 4.78 ± 1.14 in wildtype retinae compared to 1.82 ± 0.21 in retinoschisin-deficient retinae at P18 (P < 0.01)) and for Atp1b2 (r(IS/ONL) = 5.50 ± 1.50 in wildtype retinae compared to 2.50 ± 1.01 in retinoschisin-deficient retinae at P18 (*P* < 0.05)) (Fig. 4a and b, lower panel; Supplementary Figs. S5 and S6). Consistent with previous results [6], our data confirm that retinoschisin-deficiency leads to reduced Atp1a3 and Atp1b2 accumulation in the IS of photoreceptors with increasing postnatal age.

As for the retinal Na/K-ATPase, retinoschisin-deficiency also affected Kv_._2.1 and Kv8.2 localization in the photoreceptor IS (Fig. 4c and d; Supplementary Figs. S7 and S8), although the effect of retinoschisin-deficiency on Kv channel localization occurred earlier in retinal development. Statistical significance in the r(IS/ONL) of Kv2.1 between wildtype and retinoschisin-deficient mice was obtained at P7 with a r(IS/ONL) of 2.55 ± 0.37 in wildtype retinae compared to 1.40 ± 0.25 in retinoschisin-deficient retinae, (*p* < 0.01) (Fig. 4c, lower panel). Significant differences were also observed at P14 for Kv8.2 with a r (IS/ONL) of 4.63 ± 0.42 in wildtype retinae compared to 2.08 ± 0.33 in retinoschisin-deficient retinae, (*p* < 0.01) (Fig. 4d, lower panel).

### Altered retinal localization of Kv2.1 and Kv8.2 in retinoschisin-deficient mice is associated with reduced protein expression

**Fig. 5:**
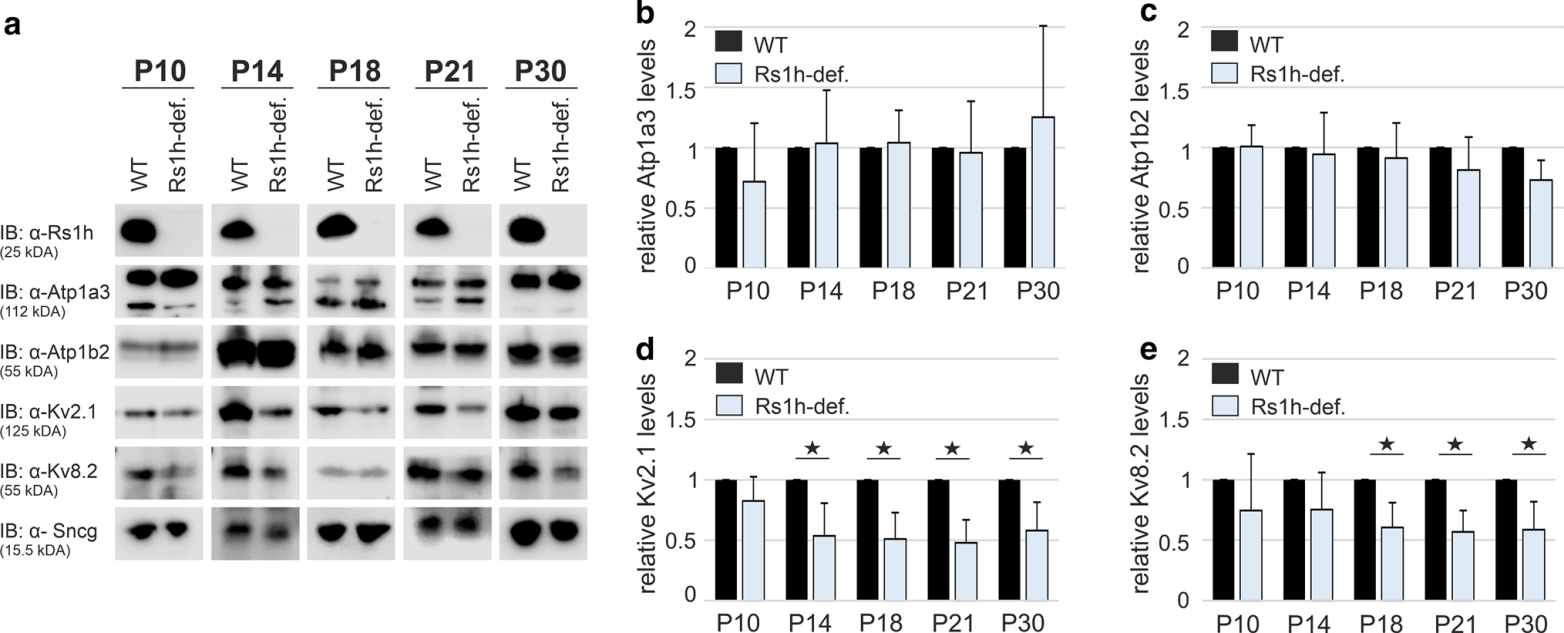
Retinoschisin-deficiency affects protein levels of Kv2.1 and Kv8.2, but not of the retinal Na/K-ATPase. **a** The total protein amount of Atp1a3, Atp1b2, Kv2.1 and Kv8.2 in retinal lysates of wildtype (WT) and retinoschisin-deficient mice (Rs1h-def.) from different postnatal stages was evaluated by western blot analysis with antibodies against Atp1a3, Atp1b2, Kv2.1, and Kv8.2. Synuclein gamma (Sncg) staining served as loading control, retinoschisin staining was used for genotype verification. Representative immunostainings from retinal lysates from P10–P30. **b**–**e** Densitometric quantification of Atp1a3 (**b**), Atp1b2 (**c**), Kv2.1 (**d**) and Kv8.2 (**e**) signals. Signals were normalized to sncg and calibrated against the signals obtained in the wildtype retinae of the corresponding postnatal stage. Data represent the mean + SD of five biological replicates. *P < 0.05, Mann–Whitney-*U* Test

In a next step, we clarified whether the spatial redistribution of Kv2.1 and Kv8.2 due to retinoschisin-deficiency is accompanied by changes in protein expression. While western blot analysis revealed no differences in the total protein amount of Atp1a3 and Atp1b2 between retinal lysates from wildtype and retinoschisin-deficient mice at all developmental stages analyzed (Fig. 5a–c), retinoschisin-deficiency leads to a statistically significant reduction (*p* < 0.05) of approximately 50% of Kv2.1 protein expression from P14 onward (Fig. 5a and d) and to approximately 40% of Kv8.2 protein levels (*p* < 0.05) from P18 onward (Fig. 5a and e). Finally, mRNA expression of *Atp1a3*, *Atp1b2*, *Kcnb1* (encoding Kv2.1)*,* and *Kcnv2* (encoding Kv8.2) in murine retinoschisin-deficient and wildtype retinae was compared at P14, P18, and P21. Quantitative RT-PCR revealed no statistically significant differences with only one exception (Supplementary Fig. S9). At P14, retinoschisin-deficiency induced a slight but statistically significant increase in the mRNA expression of *Atp1b2*. Nevertheless, this effect was not reflected in differences in protein expression of Atp1b2.

### Retinoschisin-deficiency does not influence the Kv2.1- and Kv8.2-mediated potassium ion currents

**Fig. 6:**
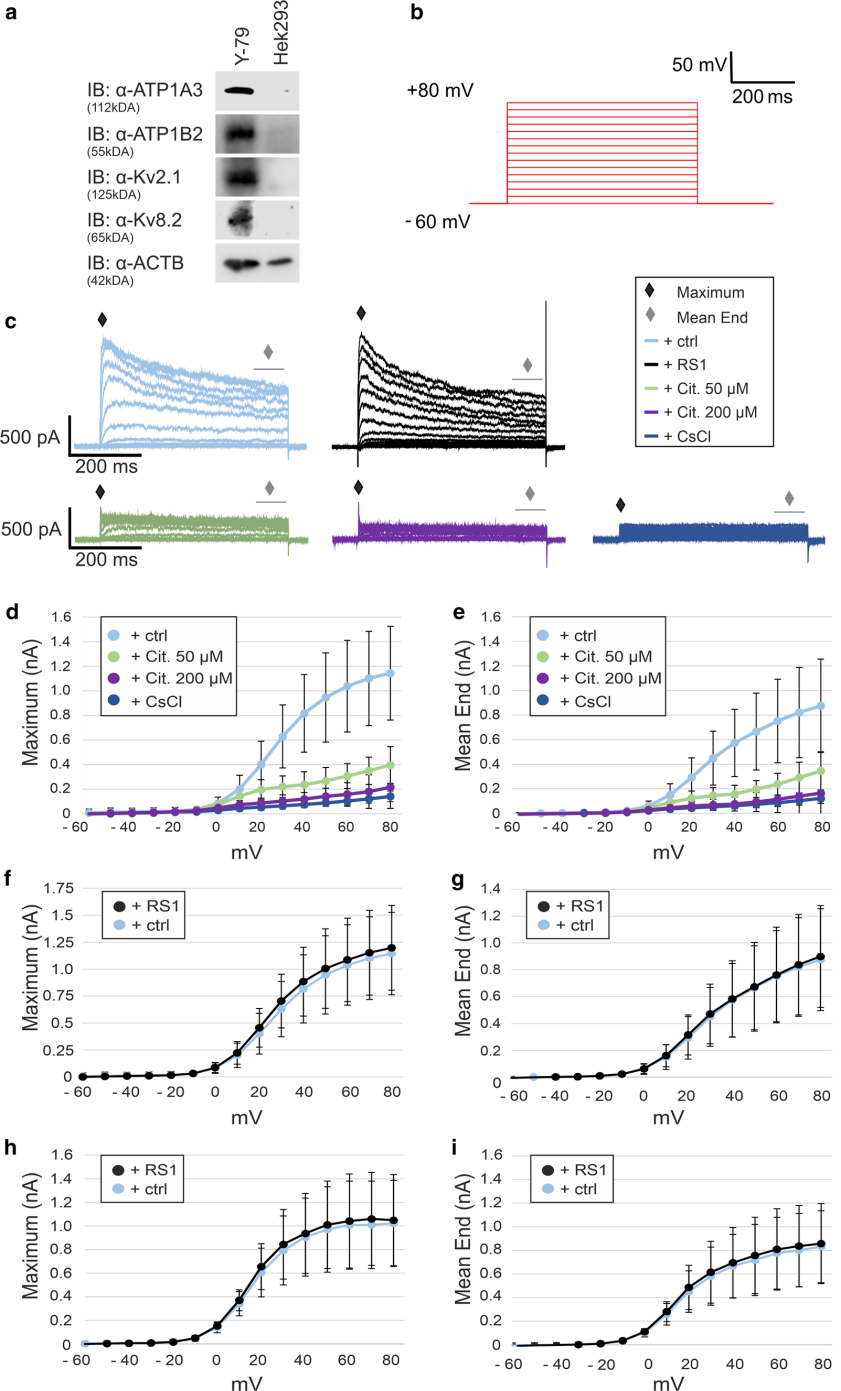
Retinoschisin has no influence on the potassium ion currents mediated by Kv2.1 and Kv8.2 in Y-79 cells. **a** Protein expression of ATP1A3, ATP1B2, Kv2.1, and Kv8.2 Y-79 and Hek293 cells, analyzed via western blot analyses with antibodies against the specified proteins. ACTB staining served as loading control. **b** Patch-clamp recording protocol of the performed analysis. The holding potential was set at − 60 mV and voltage steps between − 60 to 80 mV in 10 mV increments were applied. **c** Representative current traces of Y-79 cells incubated with control eluate (+ ctrl, light blue), retinoschisin (+ RS1, black) or with the inhibitors citalopram (+ “Cit.”, 50 µM, green; 200 µM, purple) or CsCl (+ CsCl, 130 mM, dark blue) for 24 h. The two analyzed parameters “Maximum” (black rhombus) and “Mean End” (grey rhombus), the latter defined as the average calculated at the end of the measurement (grey line) are highlighted. **d** and **e** Effect of Kv channel inhibitors CsCl (130 mM, dark blue, *n* = 11) and citalopram (“Cit.”50 µM, green, *n* = 10; 200 µM, purple, *n* = 6) Light blue circles indicate measurements without inhibitors (+ ctrl, *n* = 47). Average (± SD) voltage-gated maximum currents (**d**) or mean end (**e**) in response to voltage steps from a holding potential of -60 mV to 80 mV. **f**–**i** Effect of retinoschisin on Kv channel currents, after 24 h (**f** and **g**) and 48 h (**h** and **i**). Average (± SD) voltage-gated maximum currents (**f** and **h**) or mean end (**g** and **i**) in response to voltage steps from a holding potential of − 60 to 80 mV. Light blue circles: cells incubated with control eluate (+ ctrl, *n* = 47 for 24 h, *n* = 26 for 48 h); Black circles: cells incubated with purified retinoschisin (+ RS1, *n* = 53 for 24 h, *n* = 31 for 48 h). Statistical evaluation was performed applying the Mann–Whitney-*U* Test

In mice, the permanent potassium outward current in photoreceptors arises from continuously active Kv2.1 and Kv8.2 channels [26]. To investigate whether retinoschisin affects the potassium ion currents mediated by the Kv channels, we performed patch-clamp analyses using the retinal human retinoblastoma cell line Y-79, endogenously expressing Kv2.1, Kv8.2 (Fig. 6a), ATP1A3, and ATP1B2 [16], but not retinoschisin. The latter can be efficiently added externally as a recombinant protein [16]. Patch-clamp recordings were performed with a holding potential of -60 mV and voltage steps between -60 mV to 80 mV in 10 mV increments (Fig. 6b). The two parameters “maximum ion outflow” and “mean end” were analyzed, the latter defined as the average calculated value at the end of the measurement (Fig. 6c). To assess the specificity of our approach, we recorded currents in the presence of the K^+^ channel blocker cesium chloride (130 mM in the intracellular solution, replacing K^+^). As shown in Fig. 6c–e almost no outward current was measured, even at the highest potential of 80 mV. Furthermore, the presence of the specific Kv2 channel inhibitor citalopram [26] at 50 µM and 200 µM in the extracellular solution inhibited the currents in a dose-dependent manner, with 200 µM leading to an almost complete block of outward currents (Fig. 6c–e).

To evaluate an effect of retinoschisin on the Kv channel activity, we performed voltage-clamp recordings on Y-79 cells incubated with or without recombinant retinoschisin. Cells were incubated with control eluate and purified retinoschisin for 24 h (Fig. 6c, f and g) or 48 h (Fig. 6h and i), followed by patch-clamp analysis. After 24 h, no differences in the maximum outward current (Fig. 6f) or in the mean end (Fig. 6g) were detected between Y-79 cells incubated with or without retinoschisin. The curve progressions showing an increasing ion flow at a depolarization of − 10 mV were also comparable for both conditions (Fig. 6c, f and g). Similarly, after 48 h of incubation, retinoschisin did not affect the maximum outward current (Fig. 6h), the mean end (Fig. 6i), or the curve progressions (Fig. 6c, h and i).

## Discussion

The majority of pathologic mutations in the *RS1* gene linked to XLRS pathogenesis regularly result in an extracellular deficiency of the encoded protein retinoschisin regardless of the specific type of mutation [44]. To elucidate the consequences of the loss of retinoschisin on the developing retina and thus the pathogenesis of XLRS, the delineation of the retinoschisin interaction partners at the plasma membrane has become a longtime focus in XLRS research. The intracellular beta2-laminin and the extracellular scaffold protein alphaB-crystallin were initially described as interaction partners of retinoschisin [45]. In addition, phosphatidylserine containing lipid bilayers [46] and galactose [47] were proposed to be associated with retinoschisin. Later, a study by Shi and colleagues identified the L-type voltage-gated calcium channels (LTCC) Cav1.3 and Cav1.4 [48, 49]. Eventually, the retinal Na/K-ATPase was demonstrated to directly interact with retinoschisin by Molday and colleagues [7]. Subsequently, our group showed the specific interaction of retinoschisin with the ATP1B2 subunit, whereas the ATP1A3 subunit was exchangeable [20]. Here, we have now added another piece of the puzzle to the growing protein complex associated with retinoschisin.

By co-immunoprecipitation, we reproducibly demonstrate a physical interaction of subunit ATP1A3 of the retinal Na/K-ATPase complex with Kv channel subunits Kv2.1 and Kv8.2. Of note, iBAQ values of the complex partners reveal sub-stoichiometric amounts of Kv2.1 and Kv8.2 compared to ATP1A3 in the precipitate fractions. This may be explained by the fact that ATP1A3 shows a much more widespread distribution along the different retinal cell types/retinal layers than Kv2.1 and Kv8.2. Specifically, immunohistochemical and transcriptome analyses revealed retinal Kv2.1 and Kv8.2 protein expression predominantly in photoreceptors (www.proteinatlas.org/humanproteome/tissue/retina [50]) with a strong enrichment in photoreceptor IS [26, 28, 50, 51], and, additionally, only in a subset of bipolar cells [50, 52]. In contrast, beside a strong signal of ATP1A3 in photoreceptor IS, this protein is prominently found in the outer and inner plexiform layer, and in all retinal neurons [6, 53–55]. Consequently, quantification of the ATP1A3 complex partners in the co-immunoprecipitate from retinal lysates allows no conclusions on complex stoichiometry in the photoreceptor inner segments.

In the co-immunoprecipitation analysis targeting ATP1A3, we failed to detect Cav1.3 or Cav1.4, both putative retinoschisin interaction partners as suggested by Shi and colleagues [48, 49]. This apparent discrepancy may stem from distinct experimental approaches. While the present study targeted ATP1A3 in a co-immunoprecipitation study, the work performed by Shi and colleagues used retinoschisin as IP partner [48, 49]. Cav1.3 and Cav1.4 might thus not directly bind to the retinal Na/K-ATPase complex, but rather to retinoschisin. Of note, such a constellation could further extend the macro-complex and may even include LTCCs, known to interact with Kv2.1 in mammalian brain neurons [31].

The physical interaction of the retinal Na/K-ATPase complex with Kv channel subunits Kv2.1 and Kv8.2 was corroborated by our immunohistological co-localization studies of both Kv channels and the retinal Na/K-ATPase showing similar line-scan profiles of Atp1b2 and Kv2.1 in the IS membrane. Retinoschisin binding assays revealed that retinoschisin does not directly bind to Kv channels, thus excluding Kv channels to be linked to the retinal Na/K-ATPase via an interaction with retinoschisin. Retinoschisin-deficiency, known to result in XLRS pathology, causes an increasing mislocalization of the retinal Na/K-ATPase and the Kv channel subunits Kv2.1 and Kv8.2 during postnatal retinal development of the XLRS mouse retina. In addition, it is accompanied by a decrease of Kv2.1 and Kv8.2 protein beyond P14, whereas protein levels of the retinal Na/K-ATPase appear to be unaffected by retinoschisin deficiency. Of note, we could not observe an effect of retinoschisin on the Kv channel mediated potassium ion currents as analyzed in Y-79 cells. It remains to be shown to what extend the pathological findings of each member of the retinoschisin-retinal Na/K-ATPase-Kv channel complex contributes to initial or advanced disease development.

Consistent with our current findings, Kv2.1 and Kv8.2 were shown earlier to be highly expressed in photoreceptor IS, but absent or poorly expressed in other retinal layers [26, 27]. This agrees with our immunohistochemical localization of retinoschisin and the retinal Na/K-ATPase revealing a strong enrichment at the IS of the photoreceptors [6]. It is known that Kv channels together with the Na/K-ATPase play a major role in generating the outward dark current during phototransduction keeping the photoreceptors depolarized and driving the release of glutamate neurotransmitters [30, 33]. In addition, Kv2.1 has a structural function as it mediates spatial and functional coupling of LTCCs and ryanodine receptors in mammalian neurons [31]. Thus, Kv channels are important factors in regulating the electrophysiological integrity in the healthy retina, a process which is obviously disrupted in XLRS [1, 2]. Finally, Kv2.1 was also reported to modulate intracellular signaling [31, 32], yet another process which has been shown to be impaired in XLRS [16, 20, 56].

Similar to retinoschisin-deficient mice [56–58], recent studies of Jiang and colleagues as well as of Inamdar and colleagues reported that Kv8.2 knockout mice reveal a significantly higher apoptotic cell count, a thinner retina, and increased microglia occurrence in the subretinal space [27, 28]. Interestingly, the localization of Kv2.1 was found to be unaffected in Kv8.2 knockout mice [28]. Thus, the observed mislocalization of Kv2.1 in our retinoschisin-deficient mice is likely not a general consequence of retinal degeneration. This is supported by the fact that the mislocalization of Kv2.1 in the retinoschisin-deficient mouse was detectable as early as P7, long before photoreceptor degeneration at P14 occurs in this mouse model [56]. In contrast to Kv2.1, localization of the retinal Na/K-ATPase as well as of Kv8.2 was affected seven days later, from P14 onward. Moreover, protein amounts of Kv2.1 and Kv8.2 were found reduced beginning at P14, while alterations in protein levels of Atp1a3 and Atp1b2 were not observed in all postnatal stages analyzed. Notably, in Kv8.2 knockout mice the protein expression of the Na/K-ATPase was not found to be altered. Again, these findings argue against a general and uniform effect of photoreceptor degeneration relating to the IS membrane proteins.

RT-PCR analysis revealed no changes in mRNA gene expression as the cause for altered protein levels of Kv2.1 and Kv8.2. This is in line with a previous study by Vijayasarathy and colleagues, who used microarray-based genome-wide expression profiling and observed no mRNA expression differences for *Kcnb1* and *Kcnv2* as well as for *Atp1a*3 and *Atp1b2* in wildtype and retinoschisin-deficient retinae of P12 and P21 old mice [59]. Accordingly, the effect of retinoschisin-deficiency on Kv2.1 and Kv8.2 subunits should be attributed to a posttranslational process. One possibility may be a structural influence on complex integrity due to the absence of the Na/K-ATPase ligand retinoschisin. The integrity of a macromolecular complex is determined by its composition, i.e. the presence of specific ligands/protein binding partners [60]. The stability of the individual complex constituents is also strongly dependent on protein–protein interactions [60–62]. Retinoschisin-deficiency may disturb the formation of the macromolecular Na/K-ATPase-Kv channel complex leading to the observed distinct effects on distribution and total protein amount of the complex components. This is supported by our findings in Atp1b2-deficient mice showing that the distribution of Kv2.1 and Kv8.2 in the murine retina is altered due to the lack of the formation of the retinoschisin-Na/K-ATPase complex. Based on these observations, we speculate that the mislocalization of Kv2.1 and the distinct effects of retinoschisin-deficiency on localization and protein expression is best explained by an instability of the Na/K-ATPase-Kv-channel complex. Of note, the absence of retinoschisin and a pathologic spatial distribution of the complex partners may at least in part contribute to XLRS pathogenesis.

Our findings in the patch clamp analyses reveal no effect of retinoschisin binding on Kv channel activity in Y-79 cells. This is in line with the incapability of retinoschisin to directly bind to heterologously expressed Kv2.1/Kv8.2, as demonstrated in this study. Moreover, it disagrees with an immediate effect of retinoschisin binding to the macromolecular Na/K-ATPase complex on Kv channel activity, e.g. in a similar way as cardiotonic steroid binding to the Na/K-ATPase transactivates a variety of Na/K-ATPase associated proteins such as SRC, the IP3-receptor or the EGF-receptor, via structural alterations or trans-phosphorylation [10].

Still, retinoschisin-deficiency could have a serious impact on Kv channel activity in vivo, putatively not by the immediate consequences of a disrupted complex binding, but by the observed negative effect on Kv channel localization and absolute channel density on IS membranes.

Aberrant functionality of Kv channels or the Na/K-ATPase have been implicated in various pathological events before. It was reported that Na/K-ATPase binding to AnkB in cardiomyocytes controls the ion homeostasis via regulating the NCX activity in a local domain. Disruption of this interaction resulted in increased calcium sparks and waves, a possible mechanism for arrhythmogenesis in the AnkB syndrome [63]. Also, pathogenic mutations in *KCNB1* encoding the Kv2.1 subunit, have been identified in patients with different neurodevelopmental disorders like epilepsy or autism [64]. Further, Kv2.1 knockout mice manifest neuronal and behavioral hyperexcitability [65], as well as retinal dysfunction. Fortenbach and colleagues showed that the loss of Kv2.1 causes elevated intracellular Ca^2+^ levels due to elevated Ca^2+^ influx through cone cyclic nucleotide-gated (CNG) channels which ultimately causes rod degeneration [33]. In the brain, the defective formation of an integrin/alpha5/Kv2.1 macromolecular complex was connected to epilepsy through mechanisms such as abnormal neuronal development [66]. Finally, mutations in *KCNV2,* encoding Kv8.2, cause the retinal condition cone dystrophy with supernormal rod response (CDSSR) [67].

Taken together, our data suggest that retinoschisin may act as a crucial interaction partner of an emerging macromolecular complex at the IS of the mammalian photoreceptors by regulating the distribution and stability of the complex and its individual partners. An alteration in the spatial distribution and, consequently, the function of the complex may contribute to clinical symptoms in XLRS. Accordingly, it may be sensible to explore alternative ways to correct the secondary deficits of retinoschisin-deficiency as potential treatment options for XLRS.

## Supplementary Information

Below is the link to the electronic supplementary material.

Supplementary file1 (docx 9852 KB)

Supplementary file2 (pdf 877 KB)

Supplementary file3 (XLSX 996 KB)

Supplementary file4 (DOCX 16 KB)

Supplementary file5 (DOCX 14 KB)

Supplementary file6 (DOCX 14 KB)

Supplementary file7 (DOCX 14 KB)

## Data Availability

The data that support the findings of this study are available as Supplementary Figures and Tables and from the corresponding author on reasonable request.
