# Supplementary material for: Retinoschisin and novel Na/K-ATPase interaction partners Kv2.1 and Kv8.2 define a growing protein complex at the inner segments of mammalian photoreceptors
Source: Cell Mol Life Sci. 2022 Jul 25;79(8):448. doi: 10.1007/s00018-022-04409-9 (PMC9314279; doi:10.1007/s00018-022-04409-9)
Supplement: Supplementary file 1 — Supplementary file1 (docx 9852 KB) [file 18_2022_4409_MOESM1_ESM.docx]

**Supplementary Fig. S1:** (**a**) Co-immunoprecipitation in murine retinal lysates was performed with antibodies against Kv8.2 and with 6*His-tag as a control. Samples of input (I), flow through (FT), the last washing fraction (W) and precipitate (P, contains co-immunoprecipitated proteins) were stained with antibodies against the retinal Na/K-ATPase subunits Atp1a3 and Atp1b2, the Kv channels Kv2.1 and Kv8.2, and retinoschisin. (**b** and **c**) Co-immunoprecipitation in murine retinal lysates was performed with antibodies against (**b**) Atp1a1, (**c**) Atp1a3 and 6*His-tag as a control. Samples of input (I), flow through (FT), wash (W) and precipitate (P, contains co-immunoprecipitated proteins) were stained with antibodies against Src, Atp1a1, Atp1a3 and retinoschisin.

**Supplementary Fig. S2:** Localization of the retinoschisin-Na/K-ATPase complex and Kv2.1 / Kv8.2 in photoreceptor inner segments of the murine retina, and Atp1b2 / Kv2.1 distribution along the photoreceptor inner segment membrane. (**a** - **d**) Eye cryosections from wildtype mice (P18) were analyzed by confocal microscopy (60x magnification) after immunohistochemical stainings. Nuclei were visualized with 4',6-diamidino-2-phenylindole (DAPI) staining (blue). (**a** and **b**) Antibody stainings against Kv2.1, Kv8.2, Atp1a3, Atp1b2, and retinoschisin (green signals, respectively). (**a**) Overview images show the fine-resolution distribution of the complex partners in the murine retina. Scale bar: 20 µm. Regions of subsequent close-up images (white boxes) are shown in (**b**). (**b**) Close-up images depict localization of the complex partners in the plasma membrane of photoreceptor inner segments. Scale bar: 5 µm. (**c**) Kv2.1 (green) and Atp1b2 (red) immunohistochemistry shown in **Fig.** 2a, visualizing the position of line-scans (orange, yellow, blue, light blue) measured at the inner segment membrane. (**d**) Representative line-scans along the inner segment membrane (light blue, upper panel; orange, middle panel; yellow, lower panel; for exact positions see **Supplementary Fig. S2c**) demonstrating signal intensity profiles of anti-Kv2.1 and anti-Atp1b2 stainings. IS, inner segments; ONL, outer nuclear layer.

**Supplementary Fig. S3:** Localization of Atp1a3 (**a**) and Atp1b2 (**b**) in the retina of wildtype and Atp1b2-deficient mice at different postnatal stages. Cryosections of wildtype (WT) and Atp1b2-deficient (Atp1b2-def.) mice at P10, P14, and P18 were subjected to staining with antibodies against Atp1a3 (green) (**a**) or Atp1b2 (**b**) (red), as well as to DAPI staining (blue). Confocal microscopy was performed at 20x magnification. Scale bars: 40 µm; IS, inner segments; ONL, outer nuclear layer; INL, inner nuclear layer

**Supplementary Fig. S4:** Localization of Kv2.1 (**a**) and Kv8.2 (**b**) in the retina of wildtype and Atp1b2-deficient mice at different postnatal stages. Cryosections of wildtype (WT) and Atp1b2-deficient (Atp1b2-def.) mice at P10, P14, and P18 were subjected to stainings with antibodies against Kv2.1 (green) (**a**) or Kv8.2 (**b**) (green), as well as to DAPI staining (blue). Confocal microscopy was performed at 20x magnification. Scale bars: 40 µm; IS, inner segments; ONL, outer nuclear layer; INL, inner nuclear layer

**Supplementary Fig. S5:** Localization of Atp1a3 in the retina of wildtype and retinoschisin-deficient mice at different postnatal stages. Cryosections of wildtype (WT) and retinoschisin-deficient (Rs1h-def.) mice at P4, P7, P10, P14, P18, P21 and P30 were subjected to stainings with antibodies against Atp1a3 (green), as well as to DAPI staining (blue). Confocal microscopy was performed at 20x magnification. Scale bars: 40 µm; IS, inner segments; ONL, outer nuclear layer; INL, inner nuclear layer

**Supplementary Fig. S6:** Localization of Atp1b2 in the retina of wildtype and retinoschisin-deficient mice at different postnatal stages. Cryosections of wildtype (WT) and retinoschisin-deficient (Rs1h-def.) mice at P4, P7, P10, P14, P18, P21 and P30 were subjected to stainings with antibodies against Atp1b2 (green), as well as to DAPI staining (blue). Confocal microscopy was performed at 20x magnification. Scale bars: 40 µm; IS, inner segments; ONL, outer nuclear layer; INL, inner nuclear layer

**Supplementary Fig. S7:** Localization of Kv2.1 in the retina of wildtype and retinoschisin-deficient mice at different postnatal stages. Cryosections of wildtype (WT) and retinoschisin-deficient (Rs1h-def.) mice at P4, P7, P10, P14, P18, P21 and P30 were subjected to stainings with antibodies against Kv2.1 (green), as well as to DAPI staining (blue). Confocal microscopy was performed at 20x magnification. Scale bars: 40 µm; IS, inner segments; ONL, outer nuclear layer; INL, inner nuclear layer

**Supplementary Fig. S8:** Localization of Kv8.2 in the retina of wildtype and retinoschisin-deficient mice at different postnatal stages**.** Cryosections of wildtype (WT) and retinoschisin-deficient (Rs1h-def.) mice at P4, P7, P10, P14, P18, P21 and P30 were subjected to stainings with antibodies against Kv8.2 (green), as well as to DAPI staining (blue). Confocal microscopy was performed at 20x magnification. Scale bars: 40 µm; IS, inner segments; ONL, outer nuclear layer; INL, inner nuclear layer

**Supplementary Fig. S9.** No effect of retinoschisin-deficiency on mRNA gene expression for the genes *Atp1a3, Atp1b2*, *Kv2.1* and *Kv8.2. Atp1a3* (**a**), *Atp1b2* (**b**), *Kcnb1* (**c**), and *Kcnv2* (**d**). mRNA expression was determined in murine wildtype (WT) and retinoschisin-deficient (Rs1h-def.) retinae from different postnatal stages *via* quantitative real-time PCR. Values were normalized to synuclein gamma (*sncg*) transcript levels and calibrated against the wildtype. Data represent the mean + SD of six biological replicates, statistical evaluation was performed applying the Mann-Whitney-U Test.

**Supplementary Fig. S10.** Antibody specification. (**a**) All antibodies applied in this study were initially subjected to test antibody specificity, i. e. by staining SDS-PAGE separated and western blotted proteins from murine retinal and Hek293 cell lysates. Atp1a3, Atp1b2, retinoschisin, and Kv2.1, which are expressed in the retina but not in Hek293 [32, 68] stained protein species of the respective molecular weight, as depicted. Atp1a1 staining revealed protein species in the correct size in both murine retinal and Hek293 lysates. Kv8.2 staining in Hek293 cells was conducted with an antibody from Thermo Fisher Scientific (see Supplementary Table S3) and stained a protein species of the correct molecular weight only in Hek293 cells heterologously expressing Kv8.2. Kv8.2 staining in retinal lysates was achieved with an antibody from NeuroMab (see **Supplementary Table S3**) and also stained a protein species corresponding to the correct molecular weight. Sncg and Src antibodies also stained a protein species of the correct molecular weight (**b-e**) Full Western Blot images from Fig. 1 + Supplementary Fig S1 (**b**), Fig. 3 (**c**), Fig. 5 (**d**), and Fig. 6 (**e**).
