# Supplementary material for: Retinoschisin and novel Na/K-ATPase interaction partners Kv2.1 and Kv8.2 define a growing protein complex at the inner segments of mammalian photoreceptors
Source: Cell Mol Life Sci. 2022 Jul 25;79(8):448. doi: 10.1007/s00018-022-04409-9 (PMC9314279; doi:10.1007/s00018-022-04409-9)
Supplement: Supplementary file 2 — Supplementary file2 (pdf 877 KB) [file 18_2022_4409_MOESM2_ESM.pdf]

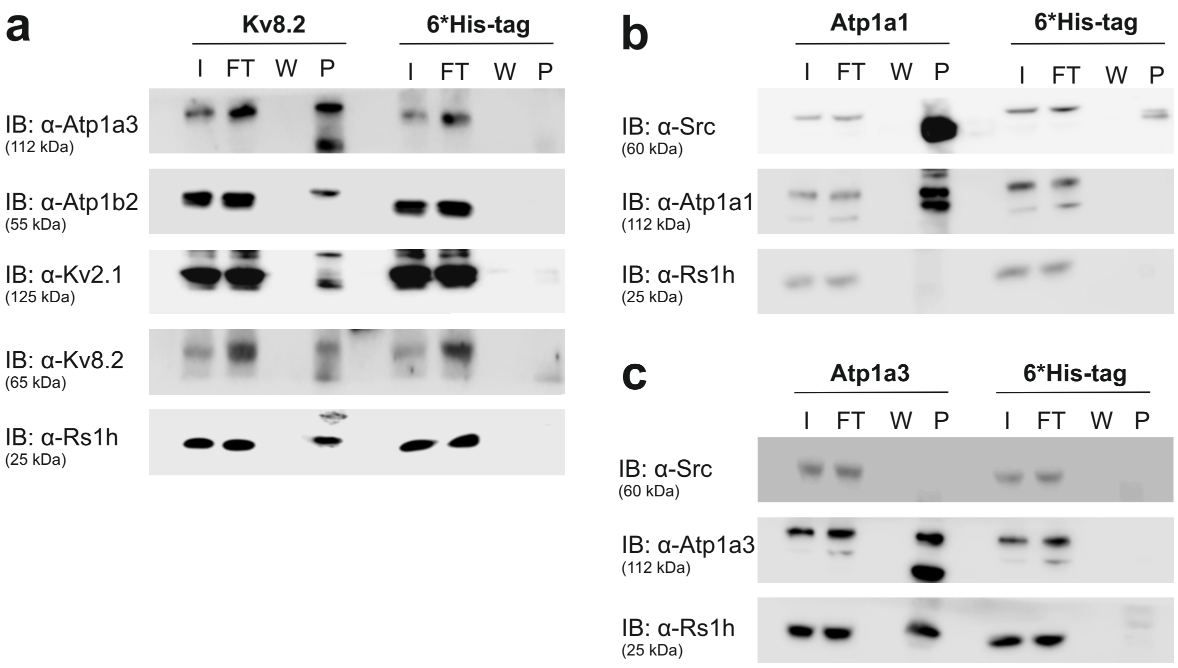

Supplemental Figure S1

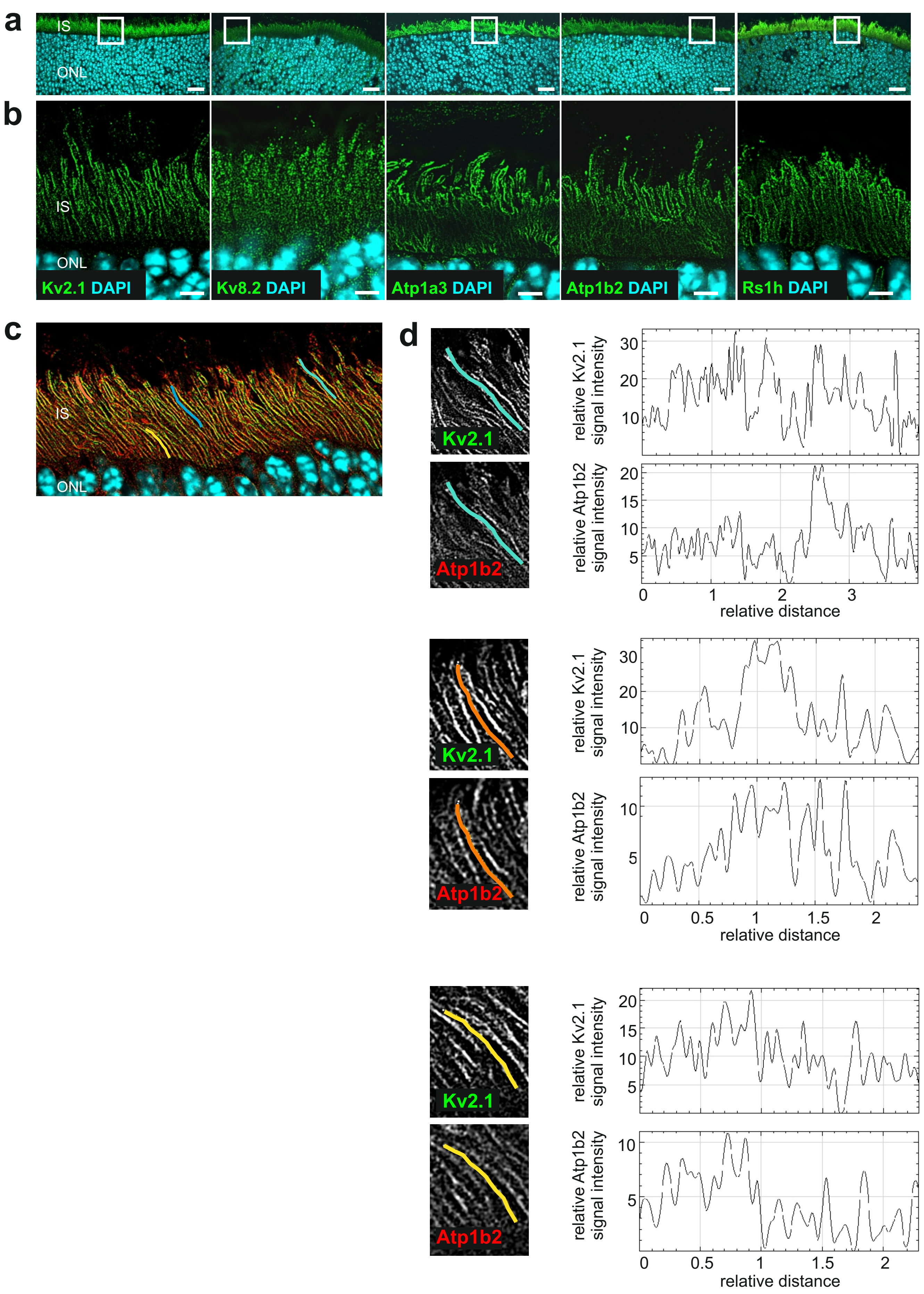

Supplemental Figure S2

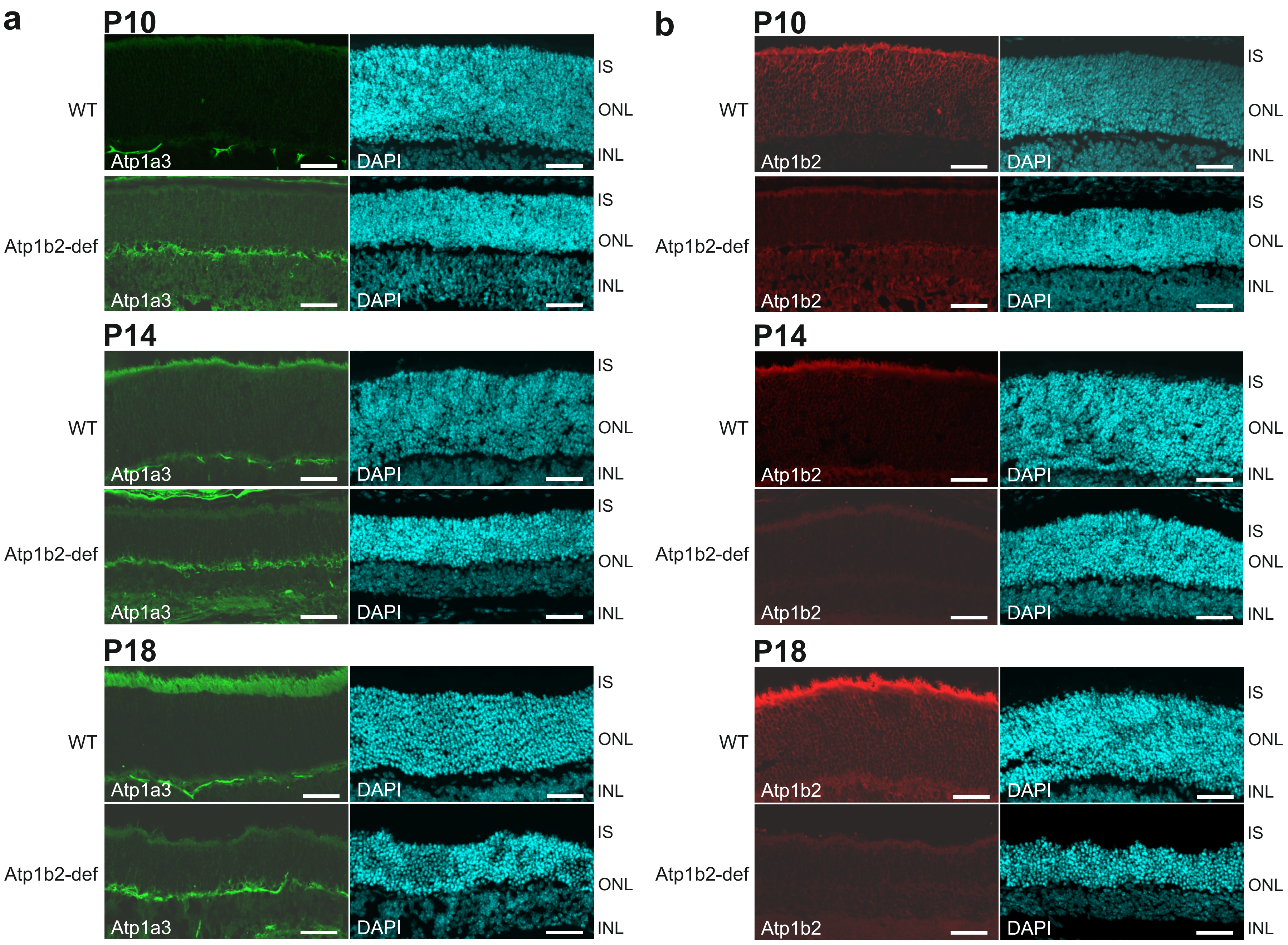

Supplemental Figure S3

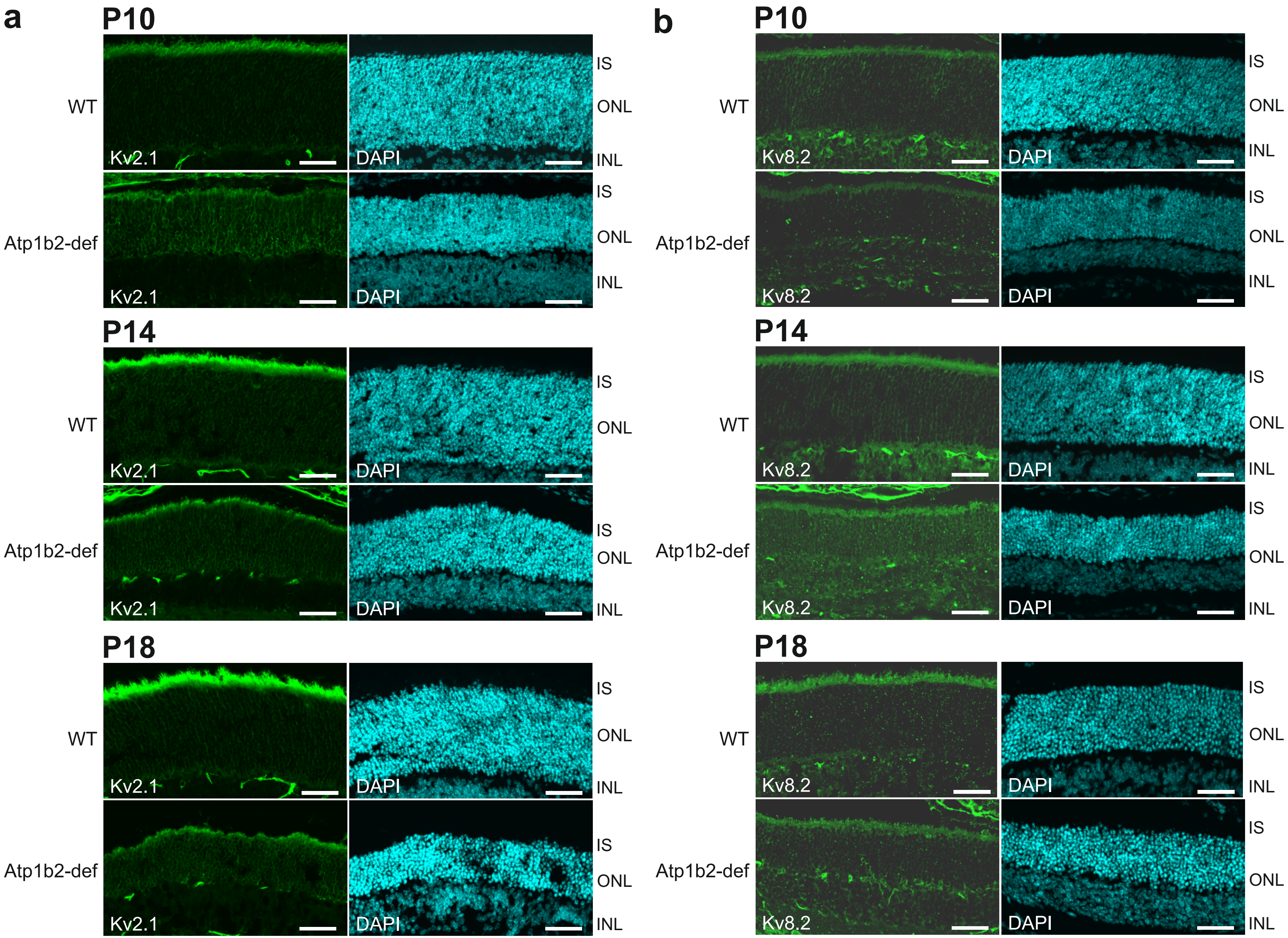

Supplemental Figure S4

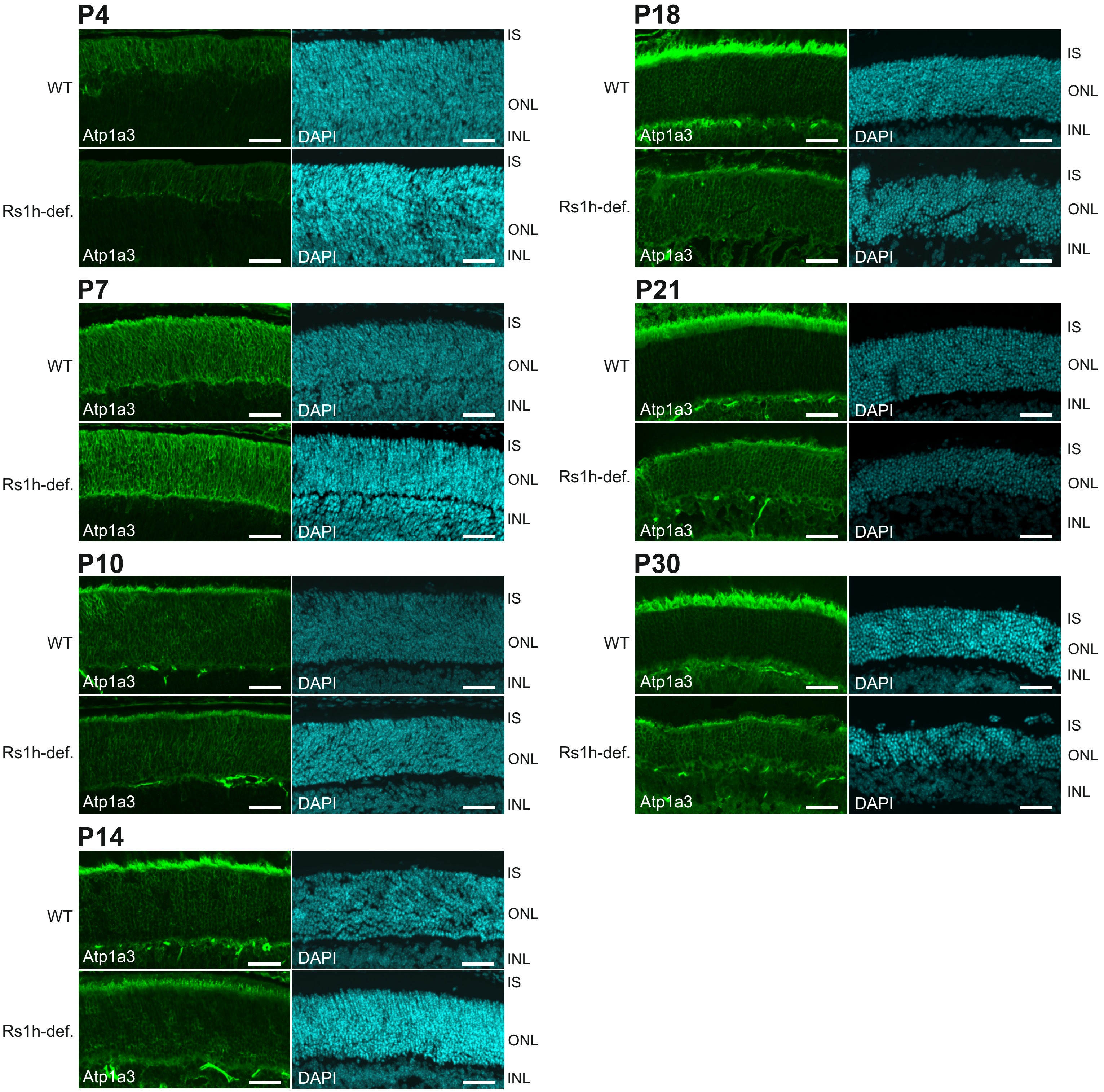

Supplemental Figure S5

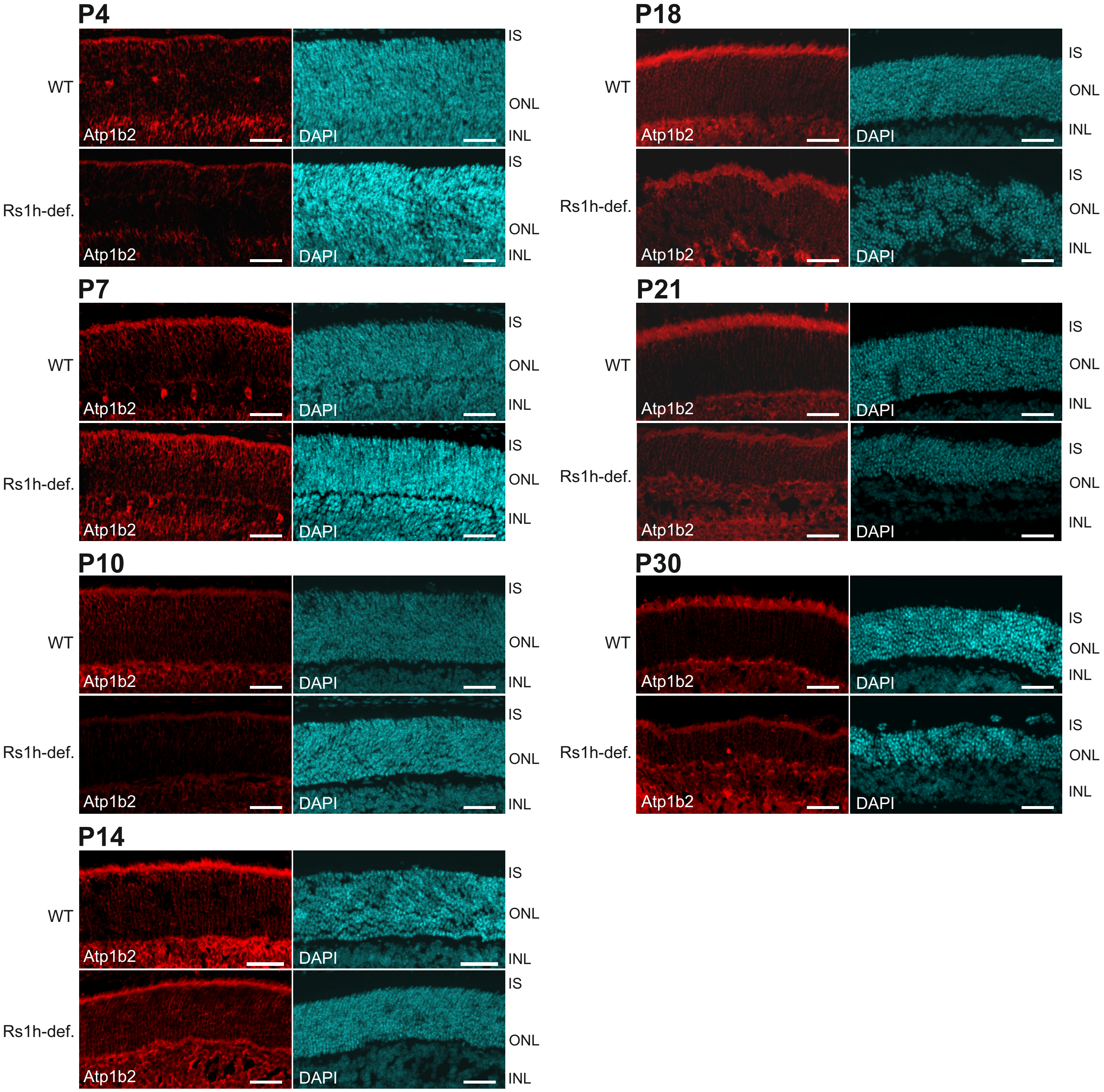

Supplemental Figure S6

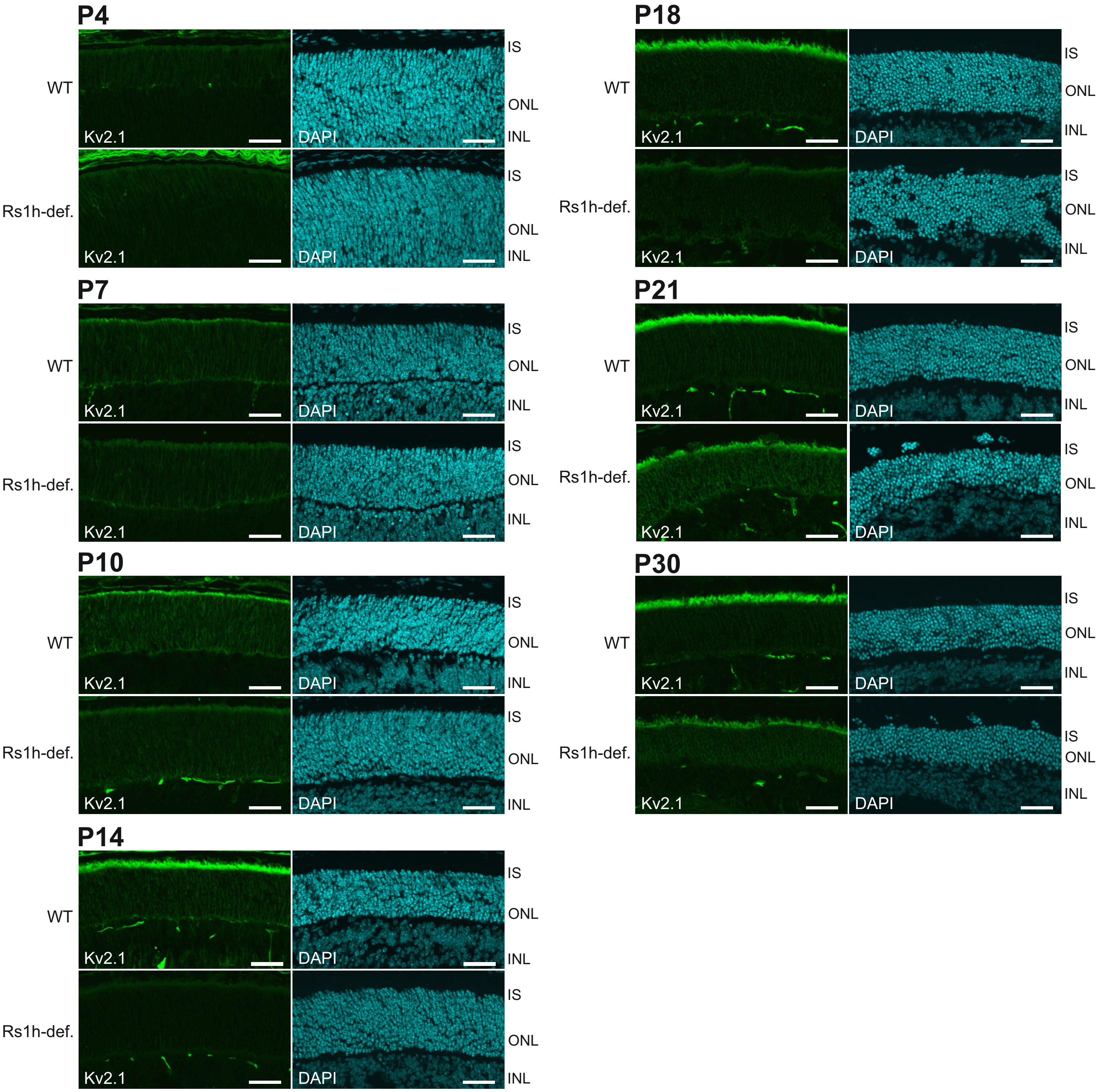

Supplemental Figure S7

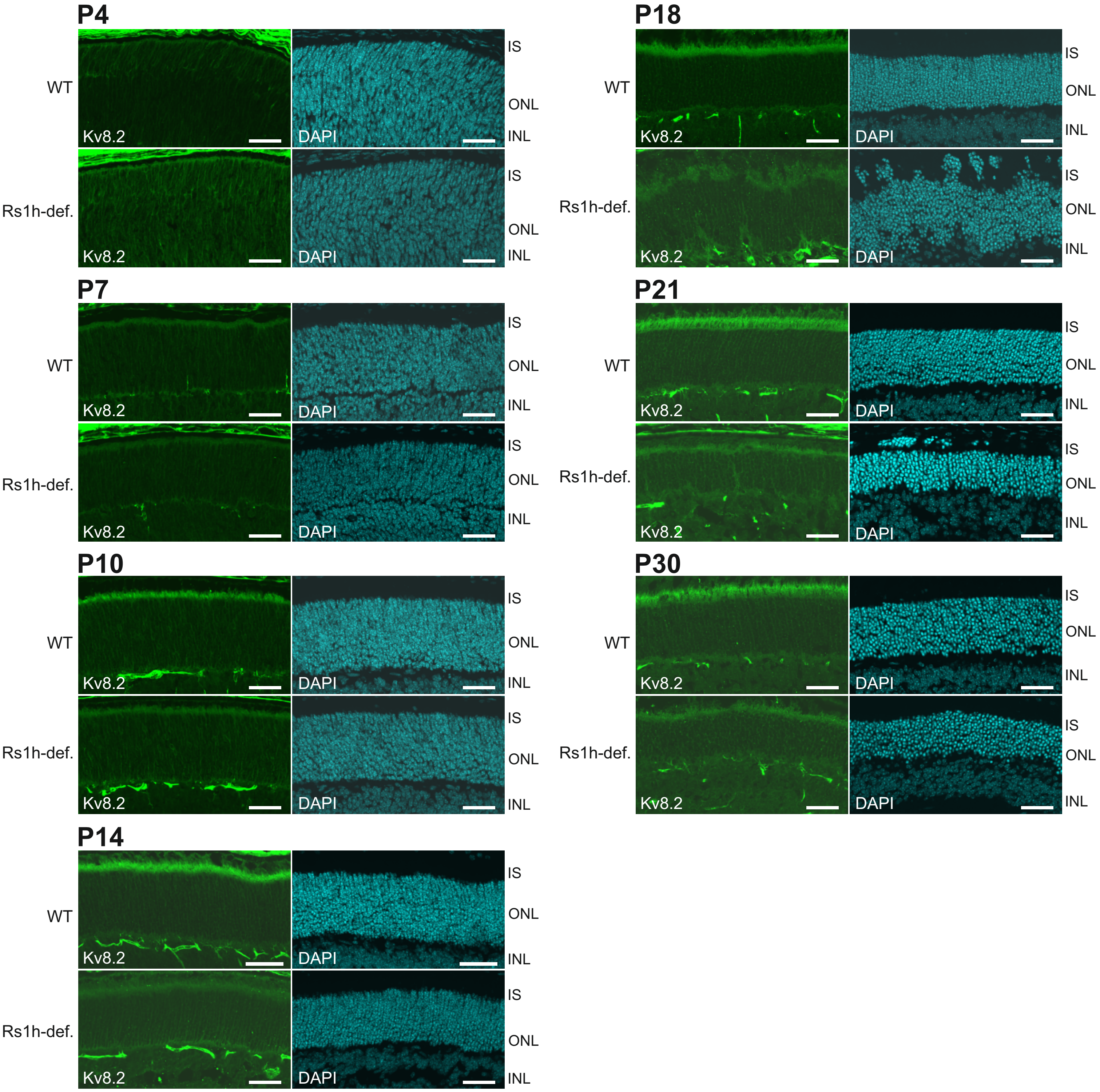

Supplemental Figure S8

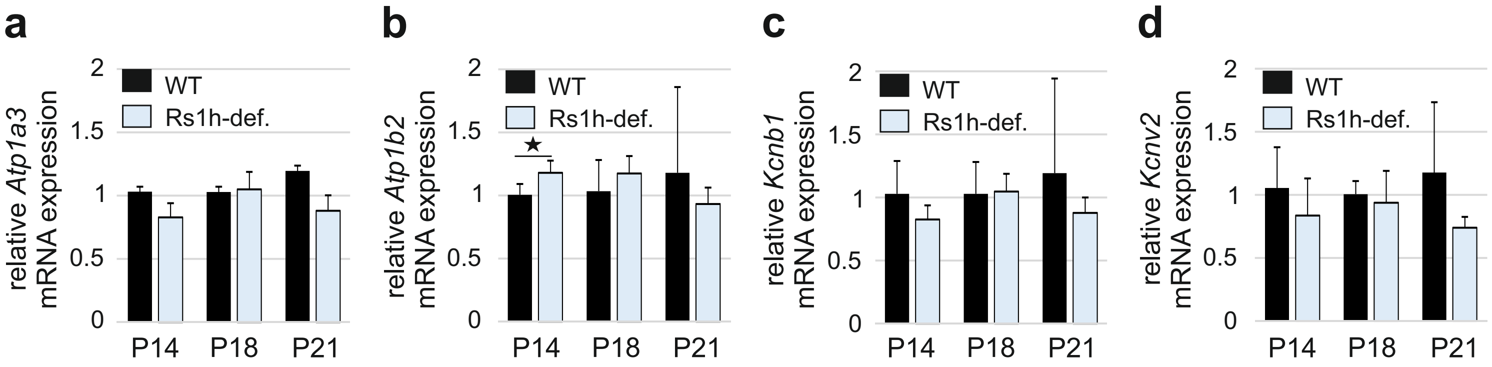

Supplemental Figure S9

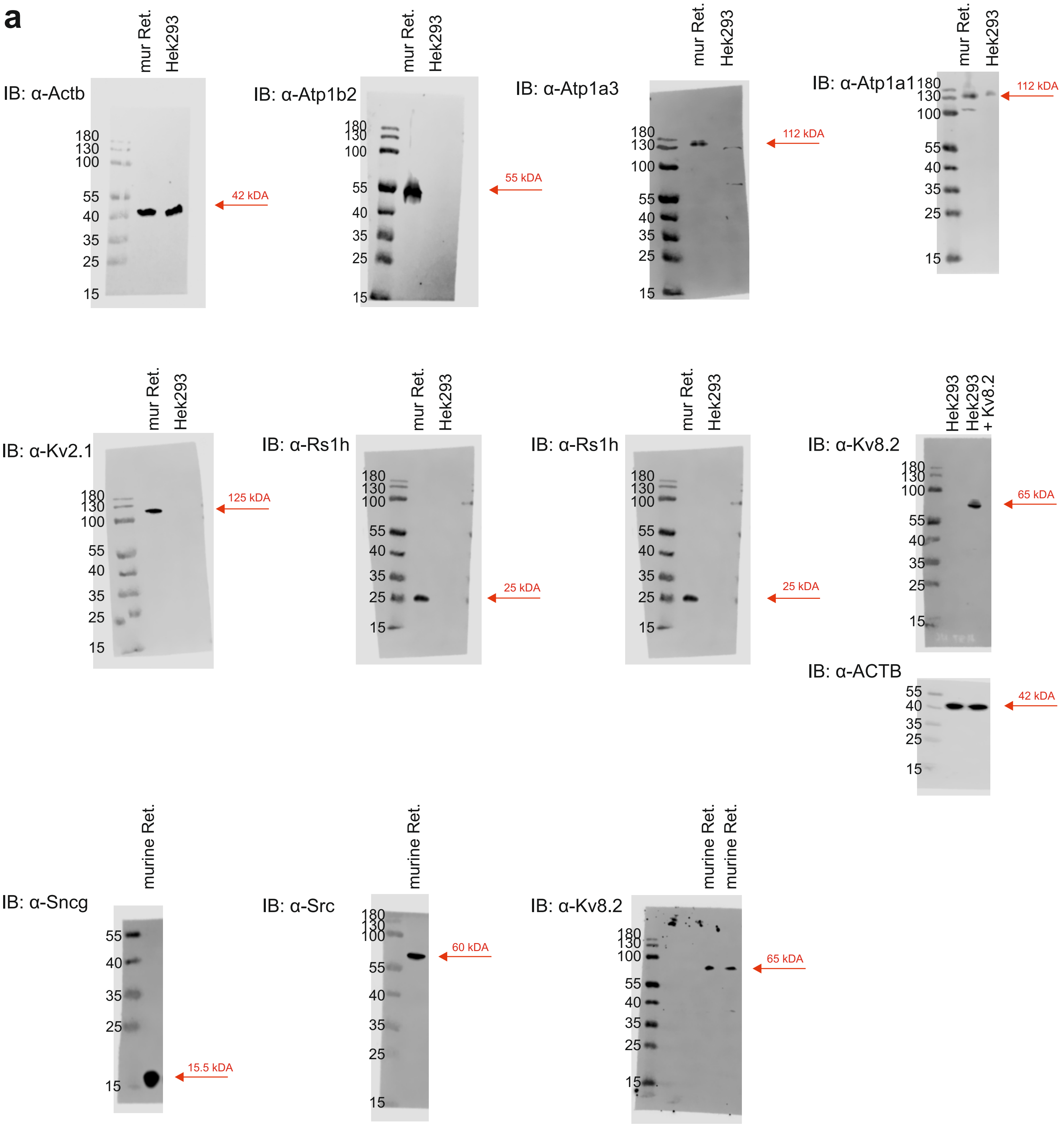

Supplemental Figure S10a

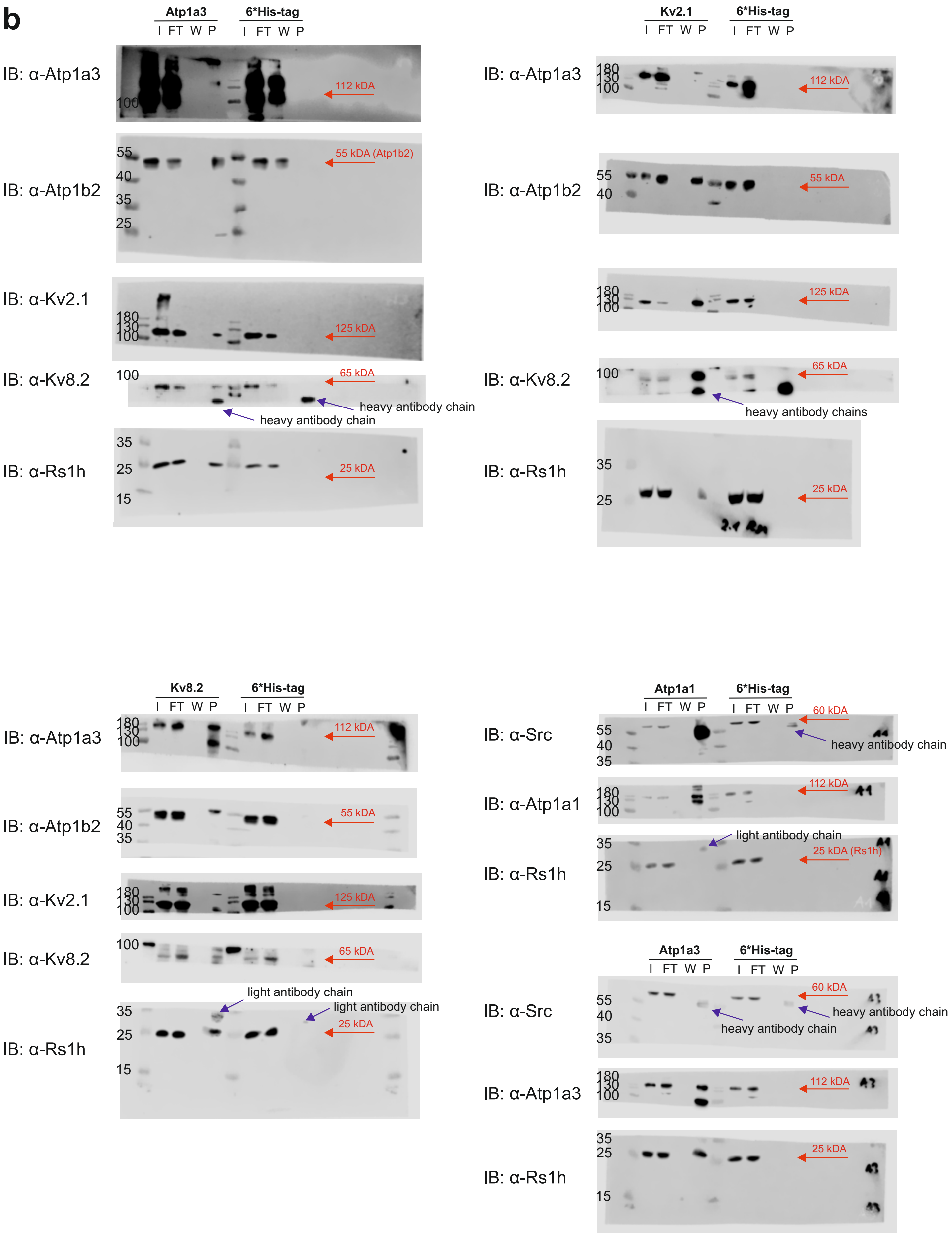

Supplemental Figure S10b

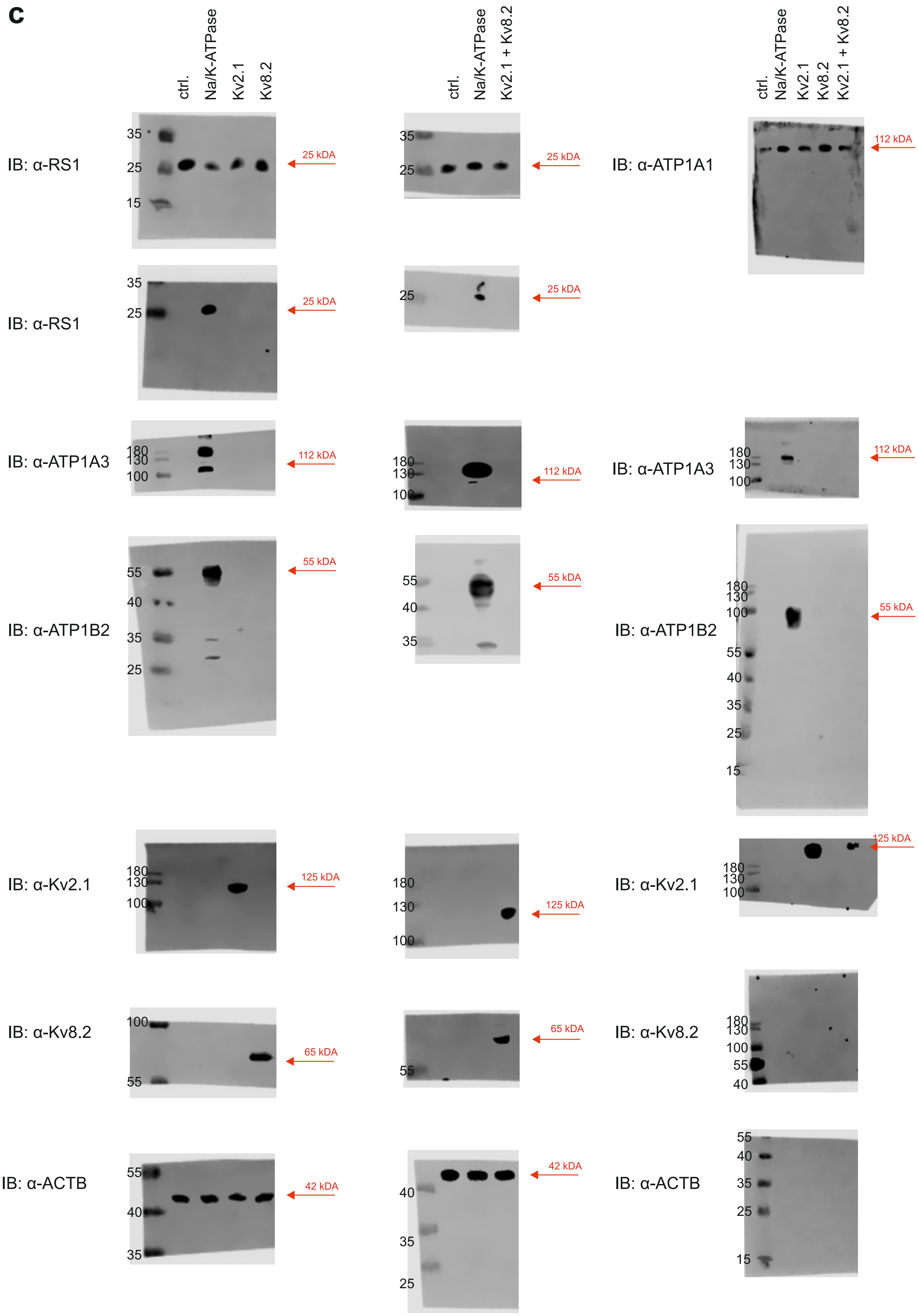

Supplemental Figure S10c

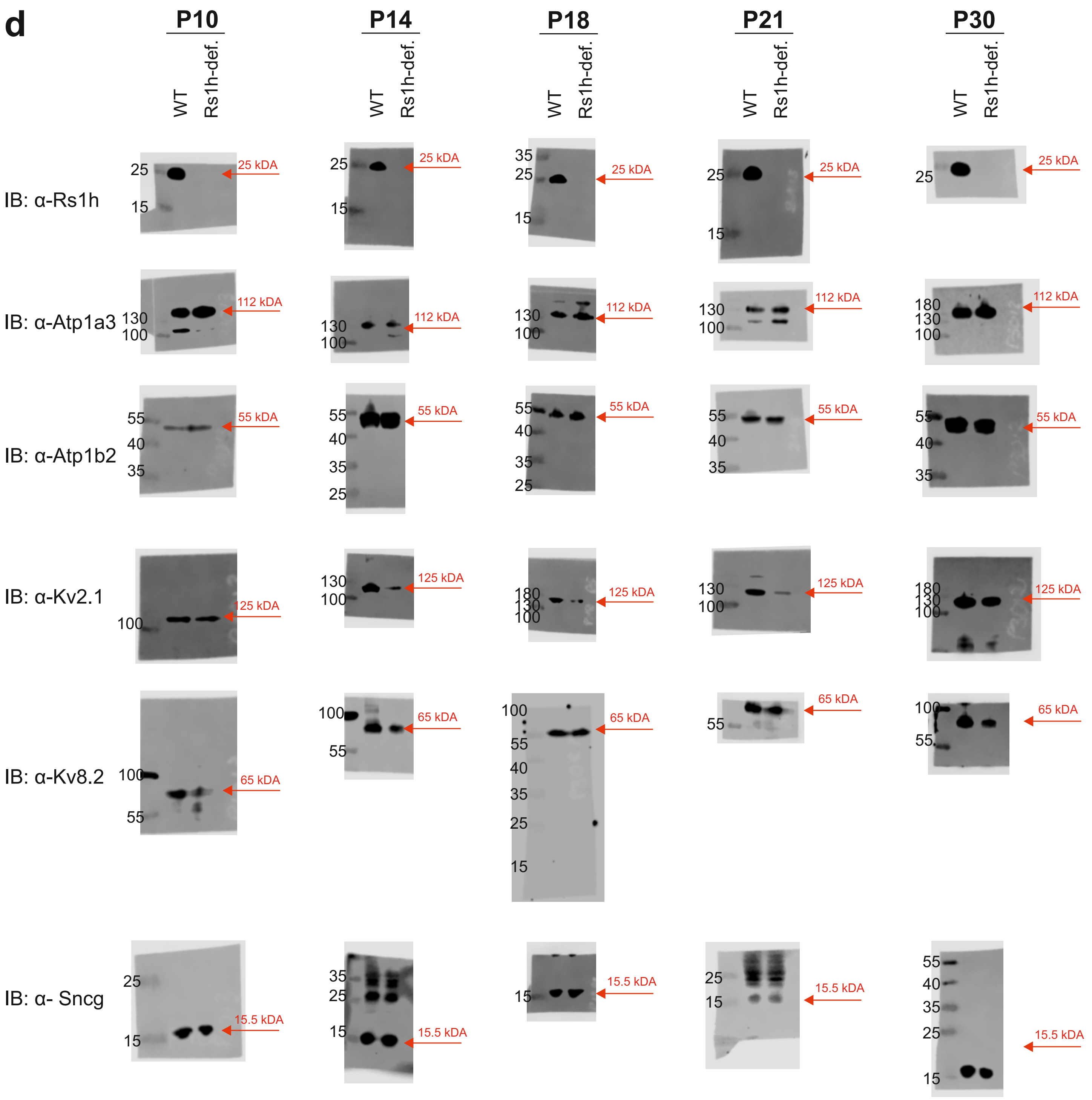

Supplemental Figure S10d

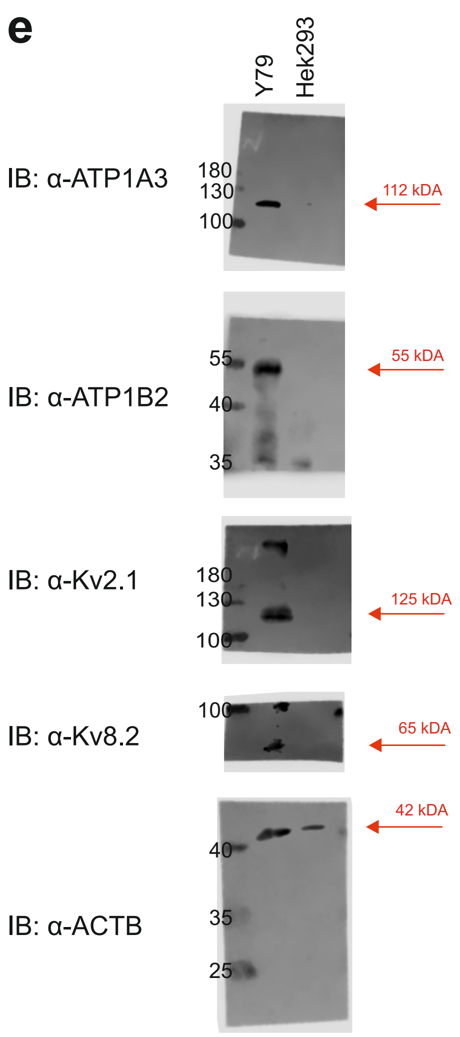

Supplemental Figure S10e
