# Supplementary material for: Retinoschisin and novel Na/K-ATPase interaction partners Kv2.1 and Kv8.2 define a growing protein complex at the inner segments of mammalian photoreceptors
Source: Cell Mol Life Sci. 2022 Jul 25;79(8):448. doi: 10.1007/s00018-022-04409-9 (PMC9314279; doi:10.1007/s00018-022-04409-9)
Supplement: Supplementary file 5 — Supplementary file5 (DOCX 14 KB) [file 18_2022_4409_MOESM5_ESM.docx]

Table S3: Primers used in the cloning of Kv2.1 and Kv8.2 expression constructs

| **Primer name** | **5’-3’- Sequence** | **Purpose** |
| --- | --- | --- |
| Kv2.1-KpnI-F | ggt acc ATG CCG GCG GGC ATG ACG AA | Amplification of the Kv2.1 coding sequence |
| Kv2.1-NotI-R | gcg gcc gcT CAG ATG CTC TGA TCT CGT GTG CTT CC |  |
| Kv8.2-KpnI-F | ggt acc ATG CTC AAA CAG AGT GAG AGG AGA C | Amplification of the Kv8.2 coding sequence |
| Kv8.2-NotI-R | gcg gcc gcC TAA TTC TCT TGT CTT GGG GTG AGC TG |  |
