# Supplementary material for: Retinoschisin and novel Na/K-ATPase interaction partners Kv2.1 and Kv8.2 define a growing protein complex at the inner segments of mammalian photoreceptors
Source: Cell Mol Life Sci. 2022 Jul 25;79(8):448. doi: 10.1007/s00018-022-04409-9 (PMC9314279; doi:10.1007/s00018-022-04409-9)
Supplement: Supplementary file 6 — Supplementary file6 (DOCX 14 KB) [file 18_2022_4409_MOESM6_ESM.docx]

Table S4: Primary antibodies used for immunohistochemical and western blotting analysis

| **Antibody** | **Host** | **Dilution and Application** | **Supplier** |
| --- | --- | --- | --- |
| Kv2.1 | Mouse | WB: 1:1000  IHC/ICC: 1:800; Fig. 2 1:400 | NeuroMab, 2B Scientific, United Kingdom, #75-014 |
| Kv8.2 | Mouse  Rabbit | WB: 1:1000  IHC/ICC: 1:800, Fig. 2 1:50  WB: 1:5000 (only used in Fig. 3a-c) | NeuroMab, 2B Scientific, United Kingdom, #75-435  Thermo Fisher Scientific, Waltham, MA, USA #PA5-76546 |
| ATP1A3 | Mouse  Rabbit | WB: 1:10000  IHC/ICC 1:1000  IHC: 1:100 (only used in Fig. 2 and Supplementary Fig. S2) | Abcam, United Kingdom, #ab2826  Proteintech, Rosemont, IL, USA, #10868 |
| ATP1A1 | Rabbit | WB: 1:10000 | Proteintech, Rosemont, IL, USA, #55187 |
| ATP1B2 | Rabbit  Rabbit | WB: 1:1000  IHC/ICC: 1:250  IHC: 1:50 (only used in Fig. 2 and Supplementary Fig. S2) | Thermo Fisher Scientific, Waltham, MA, USA, **#PA5-26279**  Proteintech, Rosemont, IL, USA, #22338 |
| RS1 | Rabbit | WB: 1:10000  IHC: 1:1000 | Professor Dr. Robert Molday, University of British Columbia, Canada |
| Src | Rabbit | WB: 1:1000 | Cell Signaling Technology, Danvers, MA, USA #6943 |
| Sncg | Mouse | WB: 1:2000 | Abnova, Taipei City, Taiwan, #H00006623-M01 |
| β-ACTB | Mouse | WB: 1:10000 | Sigma, Kanagawa, Japan, #A2228 |
