# Supplementary material for: Retinoschisin and novel Na/K-ATPase interaction partners Kv2.1 and Kv8.2 define a growing protein complex at the inner segments of mammalian photoreceptors
Source: Cell Mol Life Sci. 2022 Jul 25;79(8):448. doi: 10.1007/s00018-022-04409-9 (PMC9314279; doi:10.1007/s00018-022-04409-9)
Supplement: Supplementary file 7 — Supplementary file7 (DOCX 14 KB) [file 18_2022_4409_MOESM7_ESM.docx]

Table S5: KiCqStart® probe assays used for mRNA expression analyses

| **Probe name** | **Sense-Primer** | **Anti-sense Primer** | **5’-3’- Probe Sequence** |
| --- | --- | --- | --- |
| KCNB1  NM_008420 | CCCTACTACGTCACCATC | GGAAGATCTGGACCACAC | (6FAM)ACAGAATCCAACAAGAGCGTGC(OQA) |
| KCNV2  [NM_183179.1](http://www.ncbi.nlm.nih.gov/nuccore/NM_183179.1?report=GenBank) | atcttctccttctctgcagc | ggtacatgtctccatagccc | (6FAM)tggtggtgggccgcggtaagca(OQA) |
| ATP1A3  [NM_001290469.1](https://www.ncbi.nlm.nih.gov/nuccore/NM_001290469.1) | tcctactttgtcatcctggc | tctgctcataagtccactgc | (6FAM)cggaaacctggtgggcatccggc(OQA) |
| ATP1B2  NM_013415.5 | gagtcgggagttttctag | aagggcatctcattcataa | (6FAM)tcactcagcgacagaggacttg(OQA) |
| Sncg  ([NM_011430.3](https://www.ncbi.nlm.nih.gov/nuccore/NM_011430.3)) | ctgaaaacatcgtggtcacc | cttgctctttggcttcttgg | (6FAM)cggggtggtgcgcaaggaggact(OQA) |
